# Benchmarking the Taxonomic Resolution of Fish eDNA Metabarcodes Against COI Barcodes

**DOI:** 10.1111/1755-0998.70069

**Published:** 2025-10-30

**Authors:** Eliot Ruiz, Thomas Lamy, David Mouillot, Jean‐Dominique Durand

**Affiliations:** ^1^ MARBEC Univ Montpellier, IRD, IFREMER, CNRS Montpellier France

**Keywords:** barcode index number, clustering, DNA metabarcoding, environmental DNA, fish metabarcode, taxonomic resolution

## Abstract

Even though environmental DNA metabarcoding is revolutionizing biomonitoring, many critical steps remain unstandardized, leading to arbitrary choices, particularly regarding the selection of metabarcode, clustering method and similarity threshold, among others. Additionally, these studies were hindered by biases resulting from the presence of mislabeled sequences in international databases such as GenBank and the lack of explicit definitions for taxonomic resolution. To address these issues, we developed a robust framework to compare the performance of 22 metabarcodes derived from the same mitogenomes (all available for Actinopterygians in NCBI) against a standardized taxonomic baseline based on COI Barcode Index Numbers (BINs). This framework allows for the separate quantification of over‐splitting (splitting the same taxon/BIN) and over‐merging (merging different taxon/BIN). Comparison of OTUs obtained with multiple *de novo* clustering methods to BINs confirmed the metabarcode ranking based on error sums. Although each metabarcode exhibited varying sensitivities to over‐merging or over‐splitting errors, the clustering threshold emerged as the most important factor influencing biodiversity estimates whatever the clustering method. This led us to propose optimal thresholds for each metabarcode to delineate taxonomic levels (metabarcode gaps). Additionally, we found that taxonomic resolution varied significantly among genes, orders and community diversity, but independently of metabarcode length. Overall, the choice of metabarcode and clustering threshold should aim to minimize over‐merging or over‐splitting while ensuring accurate lower taxonomic delineations. A set of documented R functions makes this evaluation of taxonomic resolution easily applicable to any other taxonomic group for which a representative set of full genes or mitogenomes is available.

## 1 Introduction

In light of the escalating degradation of biodiversity (Carmona et al. 2021; Ceballos et al. 2015), it is essential to rapidly obtain a standardised, comprehensive and global understanding of biodiversity in order to develop management strategies aimed at minimising the pervasive effects of global change on ecosystems worldwide (Bowler et al. 2020; Jaureguiberry et al. 2022). The high cost of traditional biodiversity monitoring approaches, along with sampling and observer biases, particularly in marine environments, has impeded large‐scale efforts. This has underscored the need for standardised and accessible biodiversity monitoring methods that can be applied globally (McGeady et al. 2023; Mathon et al. 2023).

Over the past decade, DNA metabarcoding techniques, which aim to sequence the DNA from a pool of species, have emerged as the new gold standards of molecular‐based biodiversity monitoring (Xiong et al. 2022). In particular, eDNA metabarcoding focuses on environmental DNA (eDNA; Ficetola et al. 2008; Power et al. 2023), either intra‐ or extra‐cellular, present in the sampled environmental matrix (e.g., water, sediment, air; Rodriguez‐Ezpeleta et al. 2021). This approach involves multiple sequential steps including water filtration, eDNA extraction, eDNA amplification (PCR), sequencing, reads pre‐processing, reads denoising, reads clustering and taxonomic assignation (Thomsen and Willerslev 2014). Many of these steps can now be routinely performed, enabling rapid gain of insights into understudied ecosystems such as tropical and polar regions, or the deep ocean (Cote et al. 2023; Czechowski et al. 2022; de Vargas et al. 2015; Mathon et al. 2023). Consequently, eDNA holds great potential for uncovering biodiversity gradients (Bernatchez et al. 2024) and enhancing the spatio‐temporal resolution of biomonitoring (Dowell et al. 2024; Truelove et al. 2022).

Although eDNA metabarcoding is now widely used, notably to monitor marine fish communities, it remains an evolving field, with new techniques continuously being proposed and scrutinised (e.g., Yang et al. 2024). Yet, the absence of a standardised workflow hinders comparability across studies (Flück et al. 2022; Hinz et al. 2022; Zhu and Iwasaki 2023). Each step of the process presents significant challenges in sampling, molecular procedures and bioinformatics including contamination control, collection, storing, extraction, amplification, sequencing, clustering and assignment (Beng and Corlett 2020; Peres and Bracken‐Grissom 2025; Polanco et al. 2025). A key challenge is accurately identifying the optimal DNA sequence to amplify (metabarcode) and selecting a primer set that minimises PCR biases across fish taxa (Bylemans et al. 2018) to obtain a comprehensive and reliable overview of all target fish taxa occurring within a given sampled ecosystem. The length of the targeted DNA sequence is particularly crucial; it must be short enough to persist in the environment despite extracellular DNA degradation (Jo et al. 2017) while retaining sufficient DNA polymorphism to enable accurate taxonomic assignment (Ruppert et al. 2019). In addition, the reference database used for taxonomic assignment should be as complete and well‐curated as possible for the fish families and region of interest (Claver et al. 2022; Keck et al. 2023; Ruppert et al. 2019). Over the past decade, numerous bioinformatic tools have been developed to identify the optimal metabarcodes across various taxonomic groups, resulting in a wide array of primer sets available for fishes (Xiong et al. 2022). However, relatively few studies have systematically compared the performances of different fish metabarcodes within the same analytical framework, leading to limited retrospective evaluation and often an empirical selection process by users (Xiong et al. 2022).

In this study, we focused on the taxonomic resolution of metabarcodes, a critical, yet understudied factor for accurate and reliable biodiversity estimation. It has been well established that the list of taxa inferred from metabarcoding is highly sensitive to the metabarcode selection, with some metabarcodes leading to an overestimation of the actual number of taxa, while others result in underestimation (e.g., Brown et al. 2015; Collins et al. 2019; Zhang et al. 2020). Since taxonomic resolution is influenced by metabarcodes' stability in natural conditions, primers' efficiency during PCRs and metabarcodes' discriminatory power, disentangling these effects during in vivo or in vitro experiments can be challenging. Although previous *in silico* evaluations have investigated taxonomic resolution, they have often been limited to a restricted set of metabarcodes and species (e.g., Collins et al. 2019; Fontes et al. 2024; Hänfling et al. 2016; Polanco et al. 2021; Schenekar et al. 2020), employing different methodologies and definitions of taxonomic resolution, thereby complicating comparisons across studies. Here, we present a standardised *in silico* framework to evaluate the taxonomic resolution of eDNA metabarcodes using full genes or mitogenomes reference databases. We also provide a set of user‐friendly, well‐documented R functions to facilitate the reproducibility and broader application of our approach.

First, we compared metabarcodes across various genes originating from the same individual using whole mitogenomes, ensuring that any potential biases were consistently introduced across all metabarcodes within the same sequence. We specifically focused on ray‐finned fishes (Actinopterygii), a highly diverse group that is increasingly targeted in eDNA studies (Wang et al. 2021) and represented in DNA reference databases (e.g., GenBank, BOLD; Xiong et al. 2022) given their ecological and economic significance (Flandrin et al. 2024). The 26 currently available fish‐specific metabarcodes are located on four mitochondrial genes (COI, Cytb, 12S, 16S; Xiong et al. 2022). However, only a limited number of studies have provided full mitochondrial gene sequences associated with the same individual (e.g., Bonifácio et al. 2019), which is why we focused on whole mitogenomes here.

Second, the originality of our approach lies in the comparison of biodiversity estimates generated by fish metabarcodes with a standardised taxonomic reference baseline previously established for DNA barcoding, which relies on tissue‐derived DNA rather than eDNA. In vertebrates, DNA barcoding is based on a ~652 bp sequence of the COI gene (“barcode”), selected for its high taxonomic resolution (Ward et al. 2009; Andújar et al. 2018). Since barcoding predates metabarcoding (Hebert et al. 2003), the BOLD reference database (Ratnasingham and Hebert 2007) now contains COI barcodes for over 70% of morphologically described fish species, and more than 23,000 molecular taxonomic units, represented as Barcode Index Number (BIN). These BINs are defined through a single‐linkage clustering approach using a 97.8% similarity threshold further refined through Markov clustering (Refined Single Linkage or RESL), a process that is updated monthly and generally aligns well with traditional “species” delineations (Ratnasingham and Hebert 2013).

**FIGURE 1:**
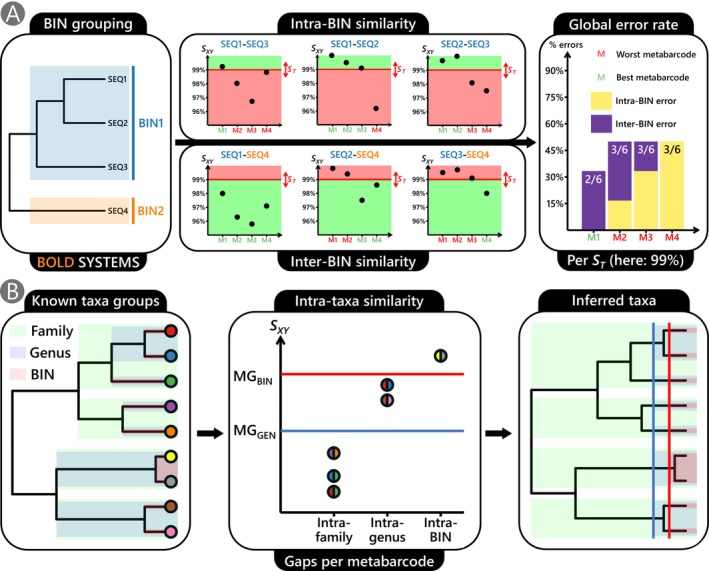
(A) Schematic representation of the framework used to compare the taxonomic resolution of metabarcodes based on BINs obtained from BOLD, which groups mitogenomes at the species level. Pairwise similarity (S_XY_) was computed between all metabarcode sequences within the same BIN (intra‐BIN analysis) and across different BIN (inter‐BIN analysis) for each metabarcode (M), and for each similarity threshold (S_T_) ranging from 90% to 99% (red box = error; green box = no error). The overall discriminatory power of each metabarcode was then assessed by summing its error rates across all possible cases for each S_T_. (B) Schematic representation of the framework used to determine optimal metabarcode gaps for distinguishing different taxonomic levels (i.e., BIN from genera, genera from families and families from orders). Using taxonomic identifications provided for each mitogenome and BINs obtained from BOLD (left plot), we computed the average S_XY_ between metabarcode sequences from different sub‐taxa within the same taxa (center plot). The optimal metabarcode gaps were defined as the mean of the first quartile of similarity values at a given taxonomic level and the third quartile at the next lower level, thereby identifying the thresholds that best separate them (MG_FAM_ and MG_ORDER_ not represented here). These cutoffs were consistently determined across all metabarcodes using all available fish mitogenomes.

Third, by using BINs as taxonomic baselines, we were able to distinguish over‐splitting errors (overestimation) from over‐merging errors (underestimation), both of which affect the estimated number of detected species compared to BINs. An over‐splitting occurs when two individuals belonging to the same BIN (i.e., intra‐BIN) are incorrectly classified as different because the pairwise similarity (*S*_*XY*_) for a given metabarcode falls below (*S*_*XY*_<*S*_*T*_) the similarity threshold denoted *S*_*T*_. Conversely, an over‐merging occurs when two individuals from distinct BINs (i.e., inter‐BIN) are erroneously considered the same because *S*_*XY*_≥*S*_*T*_ for a given metabarcode (Figure 1; Figures S2 and S3 for examples). While this comparative approach provides an unbiased estimate of the maximal discriminatory power of each metabarcode, it is only applicable when sequences are already assigned to a BIN (or species). However, because natural eDNA sequences are unknown, they must first be grouped based on sequence similarity, which may introduce biases. To account for this, we also compared OTUs (Operational Taxonomic Units) generated *de novo* (i.e., independently of any reference database) using various clustering techniques to the BIN baseline.

While both types of comparison to the BINs aim to identify the optimal clustering similarity threshold (*S*_*OPT*_), we also evaluated the best thresholds allowing the delineation of each taxonomic level during taxonomic assignment. We introduce the term metabarcoding gap (*MG*) to describe these thresholds, drawing an analogy to the widely used term “barcoding gap” in DNA barcoding. In addition to the BIN‐level metabarcoding gap between BIN and genera (*MG*_*BIN*_), we identified metabarcoding gaps between genera and families (*MG*_*GEN*_), and between families and orders (*MG*_*FAM*_). Establishing such fixed thresholds would be highly valuable for assigning unknown sequences to the most appropriate taxonomic level, particularly in incomplete reference databases, thereby improving the accuracy of taxon counts at different taxonomic levels (Bonin et al. 2023; Jackman et al. 2021). For instance, if an unknown sequence's best match consists of multiple sequences from the same genus, with similarities falling between *MG*_*GEN*_ and *MG*_*FAM*_, specific to the taxon and metabarcode used, one could reasonably infer that the individual belongs to the same family but corresponds to an unrepresented genus in the reference database.

The objective of the present study was to compare the resolving power and metabarcoding gaps of 20 metabarcodes suitable for fish (Table 1), as identified by Zhang et al. (2020). In addition to these 20 metabarcodes, we included the COI “barcode” to compare the resolving power of barcoding and metabarcoding on an equal footing. We used the COI barcode for comparison purposes, as its applicability for eDNA analysis remains debated since its universality raises concerns about non‐specific amplification, while its relatively long length might compromise both its environmental stability and sequencing efficiency (Collins et al. 2019). However, its extensive standardised reference database associated with a high discriminatory power makes it very attractive for eDNA studies (Andújar et al. 2018), while full mitogenomes comprising this sequence can be obtained from eDNA samples using long‐range PCR (Deiner et al. 2017) or even amplification‐free third generation sequencing (Ruiz et al. 2025). We first quantified over‐splitting and over‐merging across all metabarcodes using both intra/inter‐BIN and clustering approaches. We also evaluated whether the commonly used arbitrary thresholds adopted in many metabarcoding studies (e.g., 99% for OTUs) are the most appropriate for computing metabarcode gaps (Figure 1). Additionally, we simulated mock communities (i.e., known composition) to explicitly test for the relative performance of metabarcodes in sequencing outputs of varying abundance and diversity following OTU clustering (Figure 1). Finally, we investigated whether the taxonomic resolution estimated in the intra/inter‐BIN analysis depends on the metabarcode length, similarity threshold, metabarcode gene, or the taxonomic order to which each sequence belongs.

**TABLE 1:** Description of metabarcode names, locations and lengths according to different sources (including this study) and primers used to extract them.

Metabar‐code short name	Forward primer(s) name	Reverse primer(s) name	Gene	Study mean length	Design article length	Zhang et al. (2020) length	Primers orientation	Ambiguous primer(s)	Average alignment variability	Forward and reverse primers (5′ → 3′)	Design article
12SF1R1	12S_F1	12S_R1	12S	106	105	106	Normal	No	7.2%	For—ACTGGGATTAGATACCCC Rev—TAGAACAGGCTCCTCTAG	Kelly et al. (2014)
12SV5	Vert‐12SV5‐F1	Vert‐12SV5‐R1	12S	99	98	99	Reverse	No	7.2%	For—TAGAACAGGCTCCTCTAG Rev**—**TTAGATACCCCACTATGC	Riaz et al. (2011)
16SFD	Fish16SF	FishD‐2R	16S	204	200	203	Normal	Yes	9.2%	For—GACCCTATGGAGCTTTAGAC Rev**—**CGCTGTTATCCCTADRGTAACT	Berry et al. (2017) DiBattista et al. (2017)
Ac12S	Ac12S–F	Ac12S‐R	12S	390	385	389	Normal	No	7.2%	For—ACTGGGATTAGATACCCCACTATG Rev**—**GAGAGTGACGGGCGGTGT	Evans et al. (2016)
Ac16S	Ac16S‐F	Ac16S‐R	16S	338	330	336	Normal	No	8.0%	For—CCTTTTGCATCATGATTTAGC Rev**—**CAGGTGGCTGCTTTTAGGC	Evans et al. (2016)
AcMDB	AcMDB07‐F	AcMDB07‐R	12S	281	321	281	Normal	No	9.6%	For—GCCTATATACCGCCGTCG Rev**—**GTACACTTACCATGTTACGACTT	Bylemans et al. (2018)
Fish16S	Fish‐specific F	Fish‐specific R	16S	68	100	68	Reverse	Yes	11.3%	For—GGTCGCCCCAACCRAAG Rev**—**CGAGAAGACCCTWTGGAGCTTNAG	Shaw et al. (2016)
Fish2b/deg	Fish2bCBL Fish2degCBL	Fish2CBR	CytB	40	80	40	Reverse Normal	No Yes	16.4% 16.4%	For—GATGGCGTAGGCAAACAAGA Rev**—**ACAACTTCACCCCTGCAAAC For**—**ACAACTTCACCCCTGCRAAY Rev.**—**GATGGCGTAGGCAAATAGGA	Thomsen et al. (2012a)
FishCB	FishCBL	FishCBR	CytB	91	130	90	Normal	No	18%	For—TCCTTTTGAGGCGCTACAGT Rev—GGAATGCGAAGAATCGTGTT	Thomsen et al. (2012b)
FishF1‐R1	FishF1	FishR1	COI	652	652	Ø	Normal	No	15.5%	For—TCAACCAACCACAAAGACATTGGCAC Rev**—**ACTTCAGGGTGACCGAAGAATCAGAA	Ward et al. (2005)
L14735c/c2	L14735	H15149c H15149c2	CytB	413	Ø	413	Normal	No Yes	12.7% 13.1%	For—AAAAACCACCGTTGTTATTCAACTA Rev**—**ACTTCAGGGTGACCGAAGAATCAGAA For—AAAAACCACCGTTGTTATTCAACTA Rev**—**GCDCCTCARAATGAYATTTGTCCTCA	Burgener and Hübner (1998) Hänfling et al. (2016)
L14841	L14841	H15149	CytB	307	Ø	307	Normal	No	16.8%	For—AAAAAGCTTCCATCCAACATCTCAGCATGATGAAA Rev**—**AAACTGCAGCCCCTCAGAATGATATTTGTCCTCA	Kocher et al. (1989)
L14912	L14912	H15149	CytB	235	Ø	235	Normal	Yes	16.6%	For—TTCCTAGCCATACAYTAYAC Rev**—**GGTGGCKCCTCAGAAGGACATTTGKCCYCA	Miya and Nishida (2000)
L2513	L2513	H2714	16S	203	244	202	Normal	No	5.3%	For—GCCTGTTTACCAAAAACATCAC Rev**—**CTCCATAGGGTCTTCTCGTCTT	Kitano et al. (2007)
MiFish	MiFish‐U‐F	MiFish‐U‐R	12S	171	172	171	Normal	No	8.3%	For—GTCGGTAAAACTCGTGCCAGC Rev**—**CATAGTGGGGTATCTAATCCCAGTTTG	Miya et al. (2015)
PS1	PS1–F	PS1‐R	COI	Ø	247	247	Normal	Yes	Ø	For—ACCTGCCTGCCGTATTTGGYGCYTGRGCCGGRATAGT Rev—ACGCCACCGAGCCARAARCTYATRTTRTTYATTCG	Balasingham et al. (2018)
Minibar	Uni‐Minibar‐F	Uni‐Minibar‐R	COI	127	130	127	Normal	Yes	14.4%	For—TCCACTAATCACAARGATATTGGTAC Rev**—**GAAAATCATAATGAAGGCATGAGC	Meusnier et al. (2008)
Teleo1	teleo‐F	teleo‐R	12S	63	80	63	Normal	No	12.2%	For—ACACCGCCCGTCACTCT Rev—CTTCCGGTACACTTACCATG	Valentini et al. (2016)
Teleo2	tele02‐F	tele02‐R	12S	167	Ø	167	Normal	No	8.1%	For—AAACTCGTGCCAGCCACC Rev**—**GGGTATCTAATCCCAGTTTG	Taberlet et al. (2018)
Ve16S	Ve16s‐F	Ve16s‐R	16S	317	310	312	Normal	No	7.2%	For—CGAGAAGACCCTATGGAGCTTA Rev**—**AATCGTTGAACAAACGAACC	Evans et al. (2016)
Vert16S	Vert‐16S‐eDNAF1	Vert‐16S‐eDNAR1	16S	266	250	264	Normal	Yes	9.2%	For—AGACGAGAAGACCCYDTGGAGCTT Rev**—**GATCCAACATCGAGGTCGTAA	Vences et al. (2016)

## 2 Methods

### 2.1 Mitogenomes Acquisition and Preparation

We retrieved 11,185 complete mitogenomes of ray‐finned fishes (Actinopterygii) from the NCBI nucleotide database on April 27th, 2021, using the keywords “Actinopterygii complete genome” and “mitochondrion”. We excluded 587 taxa listed as unidentified/uncertain species, varieties, or hybrids in order to retain mitogenomes corresponding to specimens identified at the species level. Taxonomic IDs were retrieved using the custom *id_to_classification* function, which wraps the name2taxid function from the R package “taxizedb”. We also manually corrected 13 misspelled scientific names in NCBI before using the function *classification* from “taxizedb” to obtain the hierarchical taxonomic classification of each species based on its NCBI taxonomy ID. Since 5% of all sequences had incomplete taxonomic information following this step, this information was supplemented automatically using five additional databases via the custom *complete_taxonomy* function: Global Biodiversity Information Facility (GBIF), Integrated Taxonomic Information System (ITIS), Catalogue of Life, World Flora Online and World Register of Marine Species (WoRMS). Finally, to maintain consistency, we retained only original mitogenomes by removing those placed in the separate REFSEQ database after an extra review step from NCBI (“NC_” in accession number), as more than 99.5% were identical after revision. Following these steps, our dataset consisted of 7910 complete Actinopterygian mitogenomes, belonging to 3354 distinct species, spanning 400 families and 68 orders.

### 2.2 Genes Extraction and Alignment

The four genes in which fish metabarcodes are located (COI, CytB, 12S and 16S) were extracted from all complete mitogenomes downloaded using the exact position of their first and last nucleotides provided in the annotation of GenBank flat files, taking into account the circular nature of mitogenomes (custom R functions *gene_position* and *gene extraction*; Figure S1). Given that the forward primers of the L14735c/c2 metabarcode are located before the CytB gene (Xiong et al. 2022), we extracted an additional 100 bp before the starting position of the CytB gene. We discarded 1729 mitogenomes with incomplete taxonomic information or gene positions. After gene extractions, we removed 9 mitogenomes due to inconsistent gene lengths: either the 12S gene (outside 500–2000 bp) or the 16S gene (outside 1000–3000 bp), and 12 mitogenomes that were reverse‐oriented. Finally, each gene was aligned for the remaining 6160 complete mitogenomes using a chained guide tree (i.e., dendrogram) with the *AlignSeqs* from the R package “DECIPHER” (Wright 2020) to expedite the alignment of long and numerous DNA sequences. All steps were implemented within the custom function *decipher_rapid_alignment*.

### 2.3 Metabarcodes Extraction

We selected the 22 primer sets currently available for fish eDNA metabarcoding (Zhang et al. 2020) to extract all metabarcode sequences, considering that the metabarcodes Fish2b/deg and L14735c/c2 can each be amplified using two different primer sets, respectively (Table 1). Additionally, we included the FishF1‐R1 primer set (Ward et al. 2005) used in DNA barcoding to amplify ~652 bp of the COI gene. Metabarcodes and the COI barcode were extracted *in silico* using a standardized approach automated with the custom R function *metabarcode_extraction* (see also Figure S1). First, nucleotide positions were stored, and all alignment gaps were removed to facilitate primer searches. We identified all potential matching positions for both the forward and reverse primers of each primer set using the function *matchPattern* from the R package “biostring” (Pagès et al. 2021), allowing for a maximum of five primer‐template mismatches and one insertion or deletion. For cases where a primer matched multiple positions within a single gene, we evaluated all possible forward and reverse primer combinations and retained the pair yielding a metabarcode length closest to the documented mean length reported in Zhang et al. (2020) and the original reference (Table 1). If multiple sequences had similar lengths, we selected the primer pair that minimized primer‐template mismatches. Finally, once the optimal primer positions were identified for each primer set, we determined a consensus start and end position for each gene by selecting the most frequently observed positions across all sequences.

Since PS1 primers are long and highly degenerate, they could not be reliably matched to the COI gene, as previously noted by Zhang et al. (2020) in their *in silico* PCR. Additionally, the extraction of Fish2b/deg. and L14735c/c2 using all available primer sets resulted in identical sequences which led us to retain only sequences obtained with the Fish2b and L14735c primer sets. As a result, our final dataset included 19 metabarcodes along with the COI barcode. To assess the accuracy of our method, we compared our resulting DNA sequences to those obtained through manual extraction after aligning each gene in MEGA v11 (Tamura et al. 2021), using the custom function *view_metabarcodes*. This verification confirmed that our automatic method produced results highly consistent with the manual approach, but within minutes rather than hours, while also minimizing human errors and limitations associated with complex DNA alignments (e.g., FishCB, Teleo1). Finally, we excluded sequences from 71 mitogenomes in which extracted metabarcodes exhibited abnormal lengths (i.e., shorter than half of the first quartile or more than twice the third quartile) or contained more than 10% of missing nucleotides (“N”). After applying these quality controls, our final dataset comprised 19 metabarcodes and the COI barcode extracted from 6089 complete mitogenomes.

### 2.4 BIN Recovery

We first retrieved the BINs of 4478 out of 6089 complete mitogenomes by querying the Barcode of Life Data System (BOLD) using their NCBI accession number. This was achieved with the custom function *accession_2_bin* which implements the *bold_specimens* function from the R package “bold” (Chamberlain 2021). For the remaining 1611 mitogenomes, the *bold_identify* function was employed from the same package to match their FishF1‐R1 barcodes against the “COX1_SPECIES” database in BOLD. This approach enabled us to retrieve an additional 960 BINs, as their corresponding sequences in the BOLD “COX1_SPECIES” database exhibited 100% similarity with our target barcodes. In total, we successfully assigned BINs to 89% of all mitogenomes. To ensure consistency, this process was conducted within a single day (May 25th, 2021) using the same RESL‐based BIN delineation. The final dataset comprises 5438 complete mitogenomes representing 2844 species, 2669 BINs, 381 families and 68 orders.

### 2.5 Taxonomic Resolution Assessment: Intra‐BIN and Inter‐BIN Analyses

The pairwise similarity between sequences *X* and *Y* (*S*_*XY*_) was defined as the ratio of matching columns to the total alignment length, excluding terminal gaps. Given that the number of pairwise comparisons exceeded one billion, VSEARCH was employed as a computationally efficient tool for estimating genetic similarity (Rognes et al. 2016). The objective was to compute *S*_*XY*_ both between sequences belonging to different BINs (inter‐BIN analysis) and between sequences belonging to different sub‐taxa within a given taxonomic level (intra‐taxa analysis). For the intra‐taxa analysis, we focused on four taxonomic levels: intra‐BIN (sub‐taxa = individual sequences), intra‐genus (sub‐taxa = BINs), intra‐family (sub‐taxa = genera) and intra‐order (sub‐taxa = families). VSEARCH was employed without filtering criteria to efficiently compare all sequences within the same taxon using the custom function *intra_taxa_similarity*, enabling us to obtain the distribution of S_XY_ between sub‐taxa within each of the taxonomic levels (Figure 1B). Metabarcode gaps (MG) were computed as the mean between the first quartile of all mean intra‐taxa *S*_*XY*_ values at a given taxonomic level and the third quartile of all mean intra‐taxa *S*_*XY*_ values at the next lower taxonomic level (Figure 4). For the inter‐BIN analysis, VSEARCH was used within the custom R function *vsearch_pairwise_similarity* to compute the pairwise similarity between sequences exhibiting at least 90% similarity (as estimated via k‐mer presorting). We then filtered out the comparisons involving sequences belonging to the same BIN.

Based on all *S*_*XY*_ values obtained within and between BINs, we computed the proportion of over‐splitting errors (i.e., cases where sequences X and Y are in the same BIN but *S*_*XY*_<*S*_*T*_; function *intra_taxa_analysis*) and over‐merging errors (i.e., cases where X and Y belong to different BINs but *S*_*XY*_<*S*_*T*_; function *inter_taxa_analysis*). These error rates were calculated as a proportion of the total number of comparisons for a similarity threshold (“*S*_*T*_”) ranging from 90% to 99% for each metabarcode (see Introduction and Figure 1A). The global error rate of a given metabarcode was defined as the sum of its over‐splitting and over‐merging error rates across that *S*_*T*_ range.

In addition to taxonomic resolution estimates based on raw pairwise similarity, we incorporated *de novo* clustering methods from the widely used programs “MOTHUR” (Schloss et al. 2009) and “SWARM” (Mahé et al. 2015) into a new custom R function *cluster_metabarcodes_mothur_swarm*. While SWARM performs pairwise alignments directly from unaligned sequences, MOTHUR clustering relies on global similarity, which requires prior sequence alignment. Unlike other programs, SWARM uses the maximum number of differences allowed between directly connected sequences, rather than a global clustering threshold (*C*_*T*_). We therefore computed by multiplying the longest unaligned sequence length by the clustering threshold, before converting to an integer by rounding down. To ensure optimal alignments were optimal, we applied the *AlignSeqs* function from the R package “DECIPHER” (Wright 2020) with two iterations and refinement steps. Furthermore, we developed an additional R function, *clusters_per_primers_decipher*, to implement pairwise similarity‐based clustering methods. This function integrates linearized clustering (*Clusterize*) as well as all clustering algorithms available in the *IdClusters*/*Treeline* functions of the “DECIPHER” package (Wright 2024), using distance matrices derived from previously computed *S*_*XY*_ values. Both newly developed functions allow the simultaneous execution of multiple clustering methods across various distance thresholds (*S*_*T*_ = 90%–99% here) and enable efficient estimation of OTU errors compared to the BIN baseline (custom function *compute_otu_errors_metabarcodes*). These analyses can be performed within a few hours on a standard laptop, ensuring computational efficiency.

### 2.6 Statistical Analyses

To assess the effect of *S*_*T*_ and genes region (i.e., 12S, 16S, CytB and COI) on error rates obtained in the intra/inter‐BIN analyses, a linear mixed‐effects model (LMM) was employed, incorporating metabarcodes as a random intercept. The percentage of over‐splitting and over‐merging errors was used as the response variable in separate LMMs with parallel intercepts (not multiplicative to avoid collinearity). To account for non‐normally distributed residuals and heteroskedasticity, we performed a robust estimation of LMMs using the *rlmer* function from the R package “robustlmm” (Koller 2016). Model performance was evaluated using Nakagawa's marginal and conditional *R* ^2^ for mixed models (Nakagawa and Schielzeth 2013). The marginal *R* ^2^ quantified the variance explained by fixed effects alone (i.e., similarity threshold), while the conditional *R* ^2^ accounted for both fixed and random effects (i.e., metabarcode effect). Additionally, we utilized a modified function from Lockwood et al. (2021) to obtain 95% Wald confidence intervals and compute a *p*‐value from z‐statistics. Since COI barcodes were assumed to provide the highest taxonomic resolution, we used COI as the reference to statistically assess the effect of similarity (i.e., whether the slope for COI significantly differed from zero) and to evaluate gene‐specific differences (i.e., whether slopes varied among genes). In addition, using the same statistical framework but additive models and considering the COI gene as the reference baseline, we assessed the overall influence of the metabarcode length and the DNA region on taxonomic resolution. To account for variability introduced by different *S*_*T*_ and metabarcodes, we incorporated crossed‐random intercepts, ensuring a robust evaluation of their respective effects. Similarly, we evaluated the effect of nucleotide variability in the alignment of each metabarcode, quantified as the average proportion of the predominant nucleotide at each alignment position.

We then repeated the intra‐BIN and inter‐BIN analyses for all *S*_*T*_, focusing independently on the five taxonomic orders in our dataset that contained more than 100 BINs (i.e., Cypriniformes, Gobiiformes, Perciformes, Siluriformes and Tetraodontiformes). In this analysis, we included *S*_*T*_ as a random intercept, as our primary objective was to evaluate whether certain metabarcodes exhibited superior performance within a specific order. The reference group consisted of results obtained using the FishF1‐R1 barcode (COI gene) across the full dataset encompassing all orders. We first examined whether the taxonomic resolution of FishF1‐R1 deviated when applied to individual orders. Subsequently, we compared the taxonomic resolution of FishR1‐R1 within a given order to that of other metabarcodes for the same order.

## 3 Results

### 3.1 Taxonomic Resolution Assessment

**FIGURE 2:**
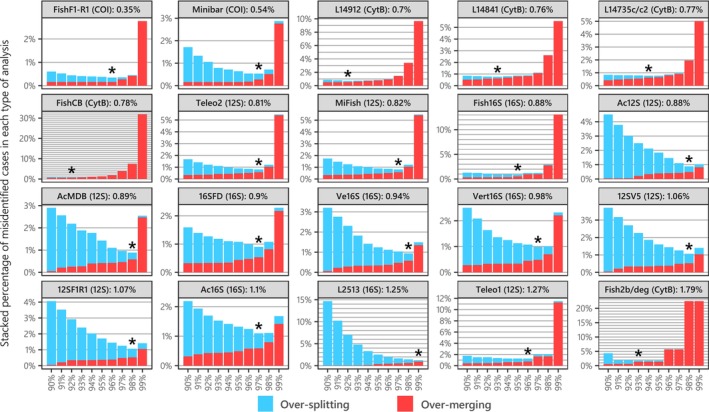
Stacked proportions of over‐splitting (red) and over‐merging (blue) errors relative to the BIN baseline as a function of S_T_, which determine the grouping of DNA sequences. Results are presented for 19 metabarcode primer sets and the FishF1‐R1 barcoding primer set. The mitochondrial gene targeted by each primer set is indicated in parentheses. For each primer set, the optimal S_T_ which minimises both error types (S_OPT_) is marked with a star, and the corresponding cumulated error rate is displayed from top left to bottom right in order of decreasing minimum cumulative proportion of over‐splitting and over‐merging errors.

**FIGURE 3:**
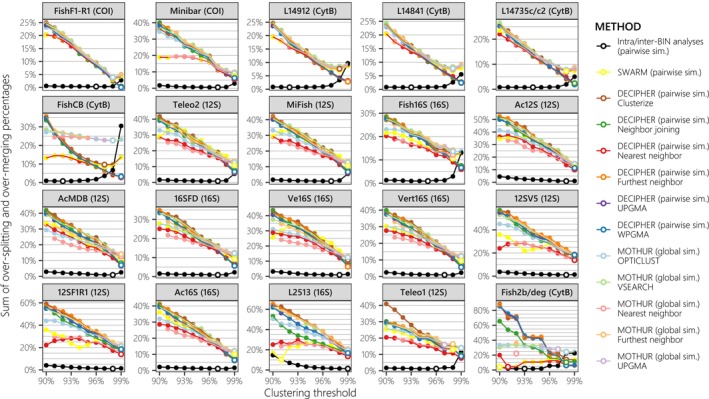
Cumulated proportions of over‐splitting and over‐merging errors in clusters generated by different methods implemented in MOTUR, SWARM and DECIPHER, plotted as a function of the clustering threshold (C_*T*_). Results are compared with those presented in Figure 2, which were derived from intra/inter‐BIN analyses. Metabarcodes are arranged in the same order as in Figure 2, based on their optimal performance in intra/inter‐BIN analyses. The mitochondrial gene targeted by each primer set is indicated in parentheses. For each primer set, the optimal C_*T*_ that minimises both over‐splitting and over‐merging errors (C_*OPT*_) is indicated with a larger point filled in white. Some are not visible due to overlapping but detailed information is provided in Table S1.

As expected, our results first show that the over‐splitting error rate increases with higher similarity thresholds (i.e., *S*_*T*_ close to 100%), whereas the over‐merging error rate, reflecting the underestimation of biodiversity relative to the actual number of BINs, declines at lower *S*_*T*_ values (i.e., *S*_*T*_ close to 90%; Figure 2). This pattern is consistent with the general principle that at high *S*_*T*_ values, sequences from the same BIN (i.e., with high *S*_*XY*_) are more likely to be erroneously identified as distinct taxa (over‐splitting: *S*_*XY*_<*S*_*T*_), whereas sequences from different BINs (i.e., with low *S*_*XY*_) are less likely to be mistakenly classified as the same taxon (over‐merging: *S*_*XY*_≥*S*_*T*_). Conversely, at lower *S*_*T*_ values, sequences from different BINs are more likely to be misclassified as the same taxon, while those from the same BIN are less likely to be erroneously split. Although this rule is well established for fish metabarcodes, our findings highlight that the magnitude of the global error rate varies significantly depending on the combination of *S*_*T*_ and the specific metabarcode used. This pattern holds true for both intra/inter‐BIN analyses (Figure 2), as well as OTUs obtained using conventional clustering methods. Notably, these clustering approaches generally exhibit higher error rates with the exception of FishF1‐R1, which maintains comparatively lower error levels (Figure 3).

For most metabarcodes, over‐splitting errors increase at a substantially faster rate with increasing *S*_*T*_ than over‐merging errors, which exhibit an almost linear increase as *S*_*T*_ decreases in both intra/inter‐BIN analyses and clustering approaches (Figure 2; Figures S7 and S8). Notably, a sharp rise in over‐splitting errors is observed across all metabarcodes when *S*_*T*_ = 99%. In many instances, this results in all sequences within the same BINs being incorrectly assigned to different taxa (over‐splitting). This misclassification typically occurs when the corresponding BINs are either represented by only two mitogenomes in our database or contain relatively dissimilar sequences (i.e., low intra‐BIN mean *S*_*XY*_; Figures S2, S5 and S6).

Second, the effect of *S*_*T*_ also varies among metabarcodes, as some of them are inherently more prone to over‐splitting or over‐merging, as demonstrated by intra/inter‐BIN analyses (Figure 2). Consequently, metabarcodes that minimize over‐splitting errors often perform worse in minimizing over‐merging errors, and vice versa (Figures S2 and S3). This pattern is exemplified by the two extreme cases: FishCB (CytB), which exhibits a higher susceptibility to over‐splitting errors and thus has a low *S*_*OPT*_ (92%), versus L2513 (16S), which shows the opposite trend, leading to a higher *S*_*OPT*_ of 99% (Figure 2). Other CytB metabarcodes share similarly low *S*_*OPT*_ compared to FishCB (92%–94%), while smaller metabarcodes such as Fish16S and Teleo1 display intermediate *S*_*OPT*_ (95%–96%). Most 12S metabarcodes behave similarly to L2513, except for a pronounced increase in over‐splitting errors at *S*_*T*_ = 99%, resulting in *S*_*OPT*_ values of 97%–98%. Additionally, other 16S metabarcodes exhibit smoother changes in over‐merging and over‐splitting error rates, despite having *S*_*OPT*_ values comparable to those of the 12S group (97%–98%).

Thirdly, the tendency of metabarcodes to be more susceptible to over‐splitting or over‐merging errors was also evident in the OTUs obtained through *de novo* clustering. However, in this case, over‐merging errors were generally more prevalent than over‐splitting errors (Figures S7 and S8). Notably, only the FishF1‐R1 barcode, CytB metabarcodes and the 3 shortest metabarcodes (Fish2b/deg., Fish16S and Teleo1) exhibited optimal clustering thresholds (*C*_*OPT*_) lower than 99% for certain clustering methods. This contrasts with the results obtained in the intra/inter‐BIN analyses (Figure 3). Overall, hierarchical clustering methods based on pairwise similarity, particularly the furthest and nearest neighbour approaches, performed best for most metabarcodes except L2513, where SWARM yielded the highest performance. In most cases, the *C*_*OPT*_ remained at 99% (Figure 3 and Table S1). Furthermore, when comparing hierarchical methods relying on global alignments (MOTHUR) versus pairwise alignments (DECIPHER), we found that global alignments generally produced the lowest over‐merging error rates at low *C*_*T*_ values. However, since their decline was more gradual with increasing *C*_*T*_, MOTHUR methods were never the most effective at high *C*_*T*_ (Table S1).

Overall, these findings indicate that the choice of an appropriate similarity threshold and clustering method tailored to the metabarcode of interest has a greater impact on taxonomic resolution than the choice of the metabarcode itself (Figures 2 and 3). For instance, in intra/inter‐BIN analyses, the maximal global error rate (*GE*_*max*_) across similarity thresholds for each metabarcode is, on average, 8.5 times higher than the minimal error rate (*GE*_*min*_). This disparity is particularly pronounced for FishCB where *GE*_*min*_ is 0.77% and *GE*_*max*_ reaches 31.80%, yielding a ratio of 41. Conversely, when considering global error rates at optimal similarity thresholds, values are much more consistent across metabarcodes (*GE*_*mean*_ = 0.93%; *GE*_*sd*_ = 0.30%). The minimal global error rate of Fish2b/deg. (*GE*_*min*_ = 1.79%) is only five times higher than that of FishF1‐R1 (*GE*_*min*_ = 0.35%), highlighting the reduced variability in error rates under optimal conditions.

### 3.2 Metabarcode Gaps

**FIGURE 4:**
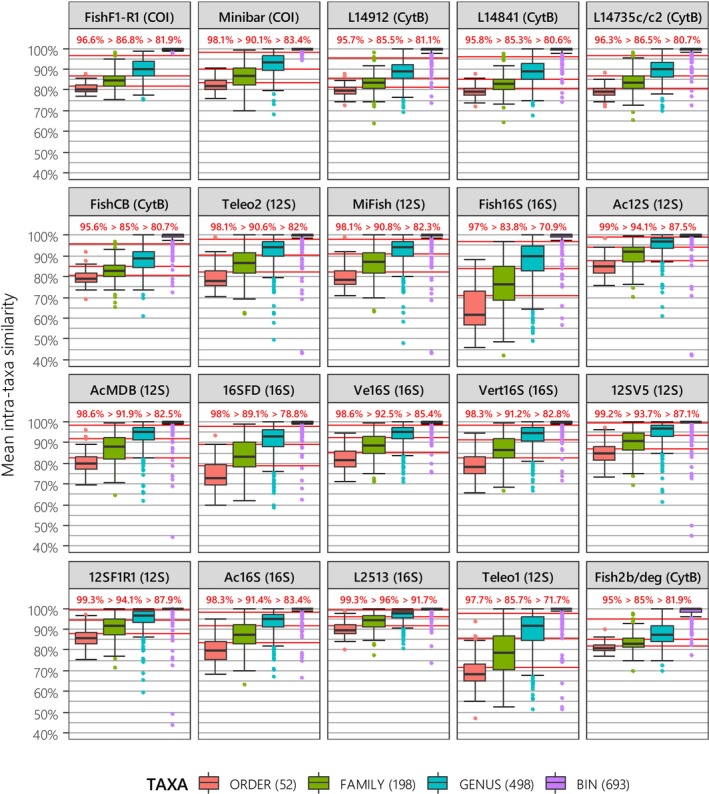
Distribution of pairwise similarity (S_*XY*_) between all possible sequence pairs from two distinct sub‐taxa within a given taxon. Metabarcodes are ordered as in Figure 2, based on their performance in intra/inter‐BIN analyses. The taxonomic levels considered include order (considering families as sub‐taxa), family (considering genera as sub‐taxa), genus (considering BINs as sub‐taxa), and BIN (considering sequences as sub‐taxa). The number of distinct taxa containing at least two sequences from two different sub‐taxa is indicated in parentheses for each taxonomic level. Metabarcodes gaps, representing the optimal delineation for each taxonomic level were computed as the mean between the first quartile of a given taxonomic level and the third quartile of the immediately lower taxonomic level. These gaps are depicted as horizontal red lines, with corresponding values displayed above them. Optimised threshold values decrease progressively, reflecting the most effective criteria for distinguishing between distinct BINs within the same genus, distinct genera from the same family, and distinct families from the same order.

Although interquartile ranges overlapped slightly in most cases, our mean intra‐taxa pairwise similarity approach (Figure 4) showed that median *S*_*XY*_ values from sequences of the same taxon but from different sub‐taxa were clearly distinguishable for all metabarcodes. The lowest overlaps were observed for the barcode FishF1‐R1, and for *MG*_*BIN*_ for most metabarcodes, suggesting that fixed thresholds will be effective for most unknown fish sequences in these cases. Similar conclusions were drawn when considering all pairwise similarities instead of means per taxa, although metabarcode gaps were slightly larger. This was probably due to the unstandardized weighting of certain taxa, particularly those well represented and highly diversified in our database (e.g., Cypriniformes comparisons = 39% of all intra‐order comparisons; Figure S9).

Another key finding was that metabarcoding gaps varied considerably among metabarcodes (Figure 4), similar to the optimal thresholds (*S*_*OPT*_). Interestingly, the mean intra‐taxa pairwise similarity was independent of the metabarcode length, except for Teleo1 (12S, 63 bp) and Fish16S (16S, 68 bp), which exhibited higher dissimilarity than other short metabarcodes such as Fish2b/deg., 12SV5, or Minibar. However, the gene location of the metabarcode appears to play a more significant role, as differences in metabarcode gaps within each gene were small (i.e., SD < 1%, except for 12S and 16S metabarcodes at family and order levels between 2% and 7%). Notably, the mean gaps for CytB metabarcodes (i.e., *MG*_*BIN*_ = 95.6%; *MG*_*GEN*_ = 85.1%; *MG*_*FAM*_ = 80.6%) were smaller than those observed for the three other genes (i.e., *MG*_*BIN*_ = 98.1%–98.6%; *MG*_*GEN*_ = 90.1%–91.5%; *MG*_*FAM*_ = 82.2%–83.4%).

A direct comparison with the BIN baseline yielded equivalent results in terms of minimal global error rate (*GE*_*min*_). Although the difference between *S*_*OPT*_ and *MG*_*BIN*_ could reach up to 3% for L14841 (*S*_*OPT*_ = 93% and *MG*_*BIN*_ = 95.8%), the *GE*_*min*_ values obtained using *MG*_*BIN*_ instead of *S*_*OPT*_ remained similar (|Δ_mean_| = 1%; |Δ_sd_| = 0.24%). Notably, *S*_*OPT*_ was always equal to or higher than the rounded third quartile of intra‐genus similarities. Compared to *S*_*OPT*_ obtained after clustering (primarily 99%), *MG*_*BIN*_ consistently fell between the lowest *S*_*OPT*_ values derived from the direct comparison of *S*_*XY*_ with *S*_*T*_ and the highest *S*_*OPT*_ values (mostly 99%) obtained after clustering. This difference arises from the additional requirement to determine whether two sequences belong to the same genus. The strong correspondence between *MG*_*BIN*_ and *S*_*OPT*_ suggests that identification errors in reference databases at each taxonomic level are likely minimal, as such errors could produce outliers that affect the quartiles used to define metabarcode gaps.

### 3.3 Statistical Analyses

**FIGURE 5:**
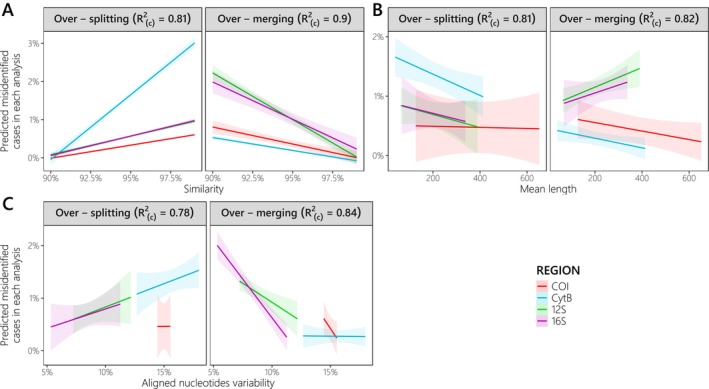
Fitted over‐splitting and over‐merging error rates based on Robust Estimates from Linear Mixed Models on each gene/region. Robust estimates from Linear Mixed Models were used to test the effect of (A) similarity threshold, (B) mean metabarcode length, and (C) nucleotide variability in metabarcode alignments on over‐splitting and over‐merging error rates for each gene. The variability induced by the metabarcode considered was randomized in all analyses, while the effect of the similarity threshold was randomized only in B and C. Nakagawa's conditional *R* ^2^ (i.e., fixed + random effects), which is displayed at the top of each plot, indicates a good model fit. The 95% confidence interval is shown around each prediction.

Our models revealed a significant linear increase of over‐splitting errors with increasing *S*_*T*_ across genes, and conversely, a decrease in over‐merging errors, as observed in intra/inter‐BIN analyses (Figure 5A). The proportion of variance explained by genes and *S*_*T*_ (i.e., fixed effects) was substantially higher than that explained by differences between metabarcodes (i.e., random effects) as indicated by the marginal *R* ^2^ values: for over‐splitting $R_{\left(m\right)}^{2}$ (i.e., $R_{\left(m\right)}^{2}=75\%$ versus $R_{\left(c\right)}^{2}-R_{\left(m\right)}^{2}=6\%$) and for over‐merging (i.e., $R_{\left(m\right)}^{2}=64\%$ vs. $R_{\left(c\right)}^{2}-R_{\left(m\right)}^{2}=26\%$). Over‐splitting error rates showed a significant decrease with increasing similarity threshold, with slopes (and intercept for CytB) significantly different from the COI gene for all other genes (Figure 5A). On the other hand, over‐merging error rates significantly increased with the similarity threshold, with slopes also significantly differing with COI for all genes, even though intercepts of COI and Cytb genes were not significantly different (Figure 5A). In summary, our results confirm that CytB metabarcodes are more sensitive to over‐splitting errors since it has the greatest slope (i.e., errors increase of 0.34% for each 1% increase of *S*_*T*_), as previously observed, while 12S and 16S metabarcodes are more prone to over‐merging errors (i.e., significantly higher intercepts). However, the error rates for 12S and 16S decreased more rapidly with increasing *S*_*T*_ compared to COI and CytB (Figure 5A).

Although the fit of LMM models with metabarcodes length and gene as fixed effects remained good (i.e., $R_{\left(c\right)}^{2}>80\%$), random effects (i.e., metabarcodes and *S*_*T*_) explained a higher proportion of the variance for over‐splitting (i.e., $R_{\left(m\right)}^{2}=21\%$ vs. $R_{\left(c\right)}^{2}-R_{\left(m\right)}^{2}=60\%$) and over‐merging (i.e., $R_{\left(m\right)}^{2}=23\%$ vs. $R_{\left(c\right)}^{2}-R_{\left(m\right)}^{2}=58\%$) error rates, respectively (see also Figure S4). The significantly decreasing trend of the over‐splitting error rate with increasing metabarcode length observed for COI (z=−2.46,p=0.014) was however, not significantly different between genes. Over‐merging error rates slightly increased with metabarcode length for 12S (z=3.68,p<0.001) and 16S (z=2.77,p=0.006) metabarcodes, and conversely for COI and CytB metabarcodes, but none of these last two trends were statistically significant (Figure 5B).

Moreover, over‐splitting errors also significantly increased with the average variability of the predominant nucleotide at each metabarcode alignment position (Table 1), with all slopes differing from that of the COI gene (Figure 5C). As for the relationship with the similarity threshold, all slopes significantly decreased with increased aligned nucleotide variability (z=−3.64,p<0.001), even though rates were this time not significantly different from that of the COI for any genes (Figure 5C). The effect of alignment variability was however less important than random effects (i.e., metabarcodes and *S*_*T*_), both for over‐splitting (i.e., $R_{\left(m\right)}^{2}=35\%$ vs. $R_{\left(c\right)}^{2}-R_{\left(m\right)}^{2}=43\%$) and over‐merging (i.e., $R_{\left(m\right)}^{2}=29\%$ vs. $R_{\left(c\right)}^{2}-R_{\left(m\right)}^{2}=55\%$) error rates, respectively.

**FIGURE 6:**
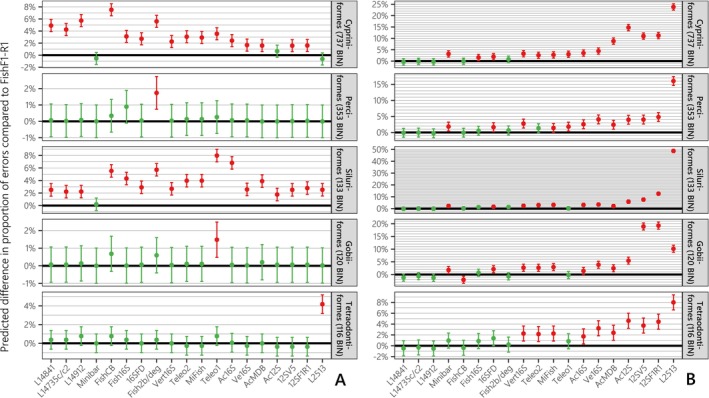
The difference in predicted over‐splitting errors (A) and over‐merging error rates (B) relative to the FishR1‐R1 barcode (bold black line) for each of the 19 metabarcodes and the five most prevalent orders of our database (number of BINs > 100). A positive percentage indicates that the metabarcode exhibits a higher error rate than FishF1‐R1 (black bold line centered on 0%) for the same order, while a negative percentage indicates a lower error rate. Red coloration denotes a statistically significant difference from 0. Green coloration indicates a non‐significant difference. The 95% Wald confidence interval is represented as an error bar around each prediction. Metabarcodes are arranged from left to right in order of increasing total predicted over‐splitting and over‐merging errors.

To evaluate whether the taxonomic resolution of metabarcodes varied among fish groups, we compared over‐splitting and over‐merging error rates of FishF1‐R1 with those of eDNA metabarcodes across the five main Actinopterygian orders in our dataset. Using FishF1‐R1 as a reference baseline was appropriate, as no other metabarcodes exhibited significantly lower error rates. However, the taxonomic resolution of FishF1‐R1 did significantly differ from the full dataset containing all mitogenomes for certain orders, specifically in terms of over‐splitting errors for Cypriniformes (i.e., +3.0%; z=5.87,p<0.001), as well as over‐merging errors for Gobiiformes (i.e., +2.5%; z=3.52,p<0.001) and Tetraodontiformes (i.e., +1.6%; z=2.25,p=0.025). LMM showed a good fit to the data (i.e., $R_{\left(c\right)}^{2}>85\%$), with fixed effects (i.e., orders and metabarcodes) explaining a much higher proportion of variance than *S*_*T*_, which was included as a random effect, both for over‐splitting (i.e., $R_{\left(m\right)}^{2}=75\%$ vs. $R_{\left(c\right)}^{2}-R_{\left(m\right)}^{2}=11\%$) and over‐merging (i.e., $R_{\left(m\right)}^{2}=83\%$ vs. $R_{\left(c\right)}^{2}-R_{\left(m\right)}^{2}=12\%$) error rates. Compared to the barcode FishF1‐R1, metabarcodes, on average, showed the largest increase of both over‐splitting and over‐merging error rates for Cypriniformes and Siluriformes (i.e., +3%–5%), while these differences were smaller for the other three remaining orders, especially for over‐splitting error rates (i.e., < 0.4%) which were relatively homogeneous among metabarcodes for these orders (Figure 6A). When considering both over‐splitting and over‐merging errors together (i.e., sum), the first three CytB metabarcodes, as well as Minibar (4th), showed the closest global error rate (*GE*) to FishF1‐R1. However, the generally homogenous over‐splitting performance of most metabarcodes for three out of five fish orders combined with their high over‐merging error rates, caused some of the best metabarcodes in the intra‐BIN analysis (e.g., Ac12S and L2513) to be ranked lower in the *GE* classification (Figure 6). Replicating the analysis across major Cypriniformes families confirmed that the same three metabarcodes yielded the lowest combined over‐splitting and over‐merging errors (Figure S10). Nonetheless, we observed marked family‐level differences, with some showing consistently low over‐splitting (e.g., Xenocyprinidae) or over‐merging (e.g., Danionidae) across all metabarcodes (Figure S10), echoing the order‐level pattern previously reported for over‐splitting (Figure 6A).

## 4 Discussion

The new framework for *in silico* taxonomic resolution assessment from full genes and mitogenomes reference databases presented in this study is designed to be both easily accessible and reproducible. This is achieved through a set of fully commented R functions and scripts for evaluating the discriminatory power of metabarcodes for any taxonomic group. First, the use of mitogenomes identified by their BINs allowed us to circumvent many taxonomic and reference database issues that have hindered previous *in silico* evaluations of primers/metabarcodes and reference databases (Marques et al. 2021). Notably, performing *in silico* PCRs on the entire EMBL database results in highly variable reference database sizes across metabarcodes (Bylemans et al. 2018; Hänfling et al. 2016; Zhang et al. 2020), which can significantly, but often unpredictably, affect species detection efficiency (Polanco et al. 2021; Schenekar et al. 2020; Somervuo et al. 2017). While recent approaches to generating curated reference databases (Jeunen et al. 2023) help mitigate *in silico* PCR biases when searching sequences with missing primer‐binding regions, using full mitogenomes remains the most reliable method for *in silico* evaluation. This approach has already been employed in four comparative studies of fish metabarcodes (Polanco et al. 2021; Schenekar et al. 2020; Somervuo et al. 2017). Second, our framework incorporates a reliable sequence extraction method that selects a consensus region across all sequences, ensuring that the extracted sequence length matches the expected metabarcode region. This approach allows for the consistent identification of the same metabarcode region across all sequences, independently of sequence‐specific variations or primer efficiencies (except for PS1). In contrast, previous metabarcode comparisons relied on alignment‐based methods (i.e., CRABS, MFEprimer, USEARCH::search_pcr and PrimerMiner) which are more prone to biases (Edgar 2010; Elbrecht and Leese 2017; Jeunen et al. 2023; Qu et al. 2012). Third, differences in how taxonomic resolution is defined across *in silico* studies complicate direct comparisons of their results. This variability might explain why four independent evaluations of more than 10 fish metabarcodes identified a different metabarcode as the most taxonomically resolutive: AcMDB07 (Shu et al. 2021), Vert‐16S (Zhang et al. 2020), MiFish‐U and Teleo2 (Collins et al. 2019), L14912 (Zhu and Iwasaki 2023). Moreover, even the most advanced methods such as neural networks (Polanco et al. 2021) have so far been unable to reliably distinguish over‐splitting from over‐merging.

**FIGURE 7:**
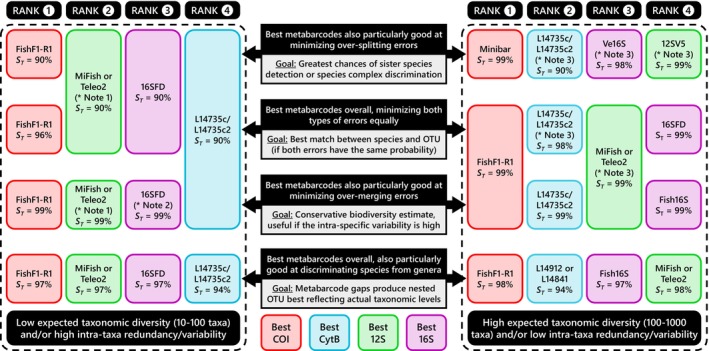
Summary of all analyses presenting the best metabarcode per gene, to guide researchers in selecting appropriate metabarcodes and similarity thresholds (ST) based on their objectives and prior knowledge of the studied community. Metabarcodes are ranked from left to right in decreasing order of relative performance (indicated by numbers at the top of the columns). The FishF1‐R1 barcode is also included in the summary, as only one COI metabarcode (Minibar) was analyzed. See Supplement S6 for details on the in silico mock community analysis that allowed the study of taxonomic resolution depending on the expected diversity and redundancy of the studied community. At a 99% threshold, Fish16S is better for Perciformes, Siluriformes, Gobiiformes and Tetraodontiformes. At a 99% threshold, Teleo1 is better for Siluriformes, Gobiiformes and Tetraodontiformes. L2513 (for CytB), Ac12S (for 12S) and 16SFD (for 16S) are more suitable for Cypriniformes.

Using our new framework with a comprehensive reference database (5438 sequences of 2844 fish species), we demonstrate that error rates varied significantly across metabarcodes (Figure 2), which is consistent with previous findings for other non‐fish specific metabarcodes (Bonin et al. 2023). Overall, the COI barcode exhibits higher taxonomic resolution than other metabarcodes in intra/inter‐BIN analyses relative to the robust OTU delineations of BOLD, as previously observed (Collins et al. 2019; Zhu and Iwasaki 2023), justifying its use as a reference baseline in LMM models. Meanwhile, Minibar and certain CytB metabarcodes display lower minimal global error rates than the most resolutive metabarcodes identified in prior *in silico* evaluations (Figure 2). Implementing multiple *de novo* clustering methods in R confirmed that ranking based on the comparison of OTU with BINs remains largely consistent, though over‐merging errors increase sharply (Figure 3 and Table S1). Surprisingly, hierarchical clustering methods perform as well as or better than centroid‐based methods (SWARM, VSEARCH, OptiClust, Clusterize; Figure 3). Previous comparisons of *de novo* clustering methods, primarily conducted on bacterial 16S/18S, have yielded conflicting rankings favoring MOTHUR's UPGMA (Schloss 2016; Wei et al. 2021), OPTICLUST (Westcott and Schloss 2017), or SWARM (Kopylova et al. 2016). In contrast, our study suggests that pairwise similarity‐based clustering (DECIPHER) performs best for fish metabarcodes (Table S1). These findings highlight that metabarcode and clustering method performances are highly dataset‐ and taxon‐dependent, reinforcing the necessity of local reference database evaluations (Dziedzic et al. 2023). Consequently, methodological choices should be tailored to the research question and community characteristics, notably by systematically performing taxonomic resolution analysis on full genes/mitogenomes local databases for the taxon of interest (Figure 7; Bonin et al. 2023).

Selecting an appropriate similarity/clustering threshold appears to be even more critical than choosing a given metabarcode or clustering method as it has a much greater impact on the relative proportion of over‐splitting and over‐merging errors (Figures 2 and 3). In cases where reference databases are incomplete or uncurated (Xiong et al. 2022), the *de novo* (i.e., reference database free) “clustering‐first” approach provides more reliable biodiversity estimates across taxonomic levels than “assignation‐first” pipelines, making it one of the key steps in metabarcoding analysis (Somervuo et al. 2017). Clustering outcomes are highly dependent on the selected similarity threshold, which should enable the clearest delineation between taxonomic levels, referred to as “m etabarcode gaps” in this study. However, the existence of universal gaps is highly debated (Edgar 2018; Jackman et al. 2021). Researchers often rely on arbitrarily chosen thresholds (Xiong et al. 2022), or inferred values from previous studies, despite differences in metabarcodes, taxa and reference databases (Kumar et al. 2020). Studies on fish diversity using traditional sampling or mock communities as a reference baseline have reported very high metabarcode gaps for MiFish (99.5%; Jackman et al. 2021) and for L14841 and 12SF1/R1 (99.8% and 99.9%; Hänfling et al. 2016). However, in this study, we highlight reduced *MG*_*BIN*_ values for these metabarcodes when considering a much greater taxonomic diversity (Figure 4). In general, the five best‐performing metabarcodes in terms of over‐merging and over‐splitting error rates (Figures 2 and 3) also exhibit the largest BIN metabarcode gaps (Figure 4). The varying sensitivities of each metabarcode to different error types can be explained by the distribution of intra‐BIN and intra‐genus pairwise similarities. Metabarcodes prone to over‐splitting (e.g., FishCB) tend to have greater dispersion of intra‐BIN similarities and lower intra‐genus similarities. In contrast, metabarcodes more susceptible to over‐merging exhibit intra‐BIN similarities close to 100% with very low dispersion around the median (e.g., L2513). The simplified metabarcode gap definition by Claver et al. (2022) relies on coefficients arbitrarily set for each taxonomic level multiplied by a metric representing the dispersion in minimum values of a boxplot, which makes it highly dependent on outliers, and therefore provides gaps at higher similarities than ours for all the taxonomic levels and metabarcodes (FishF1‐R1, MiFish and Teleo1) considered (difference: 1%–19%). Overall, each taxonomic group, from prokaryotes to mammals (Somervuo et al. 2017), along with its associated metabarcodes (Alberdi et al. 2018; Bonin et al. 2023; Edgar 2018), appears to have its own metabarcode gap. This variability strongly argues against the use of arbitrarily fixed similarity/clustering thresholds, as even slight differences can lead to substantial increases in error rates (Figures 2 and 3). Instead, metabarcode gaps could serve as an initial automated step in taxonomic assignment, followed by a necessary manual review, given that intra‐taxa similarities vary widely (i.e., see outliers in Figure 4), which is often due to misidentifications/contaminations or interspecific introgression (Claver et al. 2022). Here, we did not account for mislabeled genera, families, or orders in GenBank, as their impact is likely minimal given their rarity: at the genus level for Metazoans, mislabeling rates are typically below 1% (Leray et al. 2019).

Both statistical modelling (LMM) and mock community simulations provide valuable insights into the differences in taxonomic resolution among metabarcodes in intra/inter‐BIN analyses. First, we demonstrate that these differences are well explained by the gene targeted by each metabarcode, and by the alignment variability rather than the metabarcode length (Figure 5), which has traditionally been considered the predominant factor in previous *in silico* assessments (Collins et al. 2019; Polanco et al. 2021; Shu et al. 2021; Zhang et al. 2020; Zhu and Iwasaki 2023). Both gene identity and similarity threshold account for most of the variability observed across metabarcodes, with over‐splitting error rates increasing more rapidly with similarity for CytB metabarcodes, while over‐merging error rates are more critical for ribosomal metabarcodes, which evolve at faster rates and therefore present more variable alignments (Figure 5 and Table 1). These patterns are consistent with those observed in Figure 2. The strong consistency of metabarcode gaps across genes explains the predominant effect of genes in LMM analysis. The lowest metabarcode gaps are observed for the COI and CytB metabarcodes (except Minibar), as well as for whole COI/CytB genes intra‐family similarities (Dziedzic et al. 2023). This could be attributed to the lower evolutionary rate imposed by their conserved codon structure, which makes them less susceptible to homoplasy (i.e., convergence in sequence similarity not due to direct evolution; Meiklejohn et al. 2014). Beyond the primary effects of genes and similarity thresholds, the LMM framework also allowed us to specifically assess the relative discriminatory power of metabarcodes across the five major fish orders in our dataset, using the FishF1‐R1 barcode as a reference baseline (Figure 6). In general, over‐merging rates varied more across metabarcodes than across orders, whereas over‐splitting rates are more pronounced for Siluriformes and Cypriniformes (Figure 6), with the latter showing the same pattern across its major families despite sometimes large differences among them (Figure S10). Sequences from Cypriniformes and Siluriformes do not exhibit any single distinguishing characteristic compared to the other three orders, in terms of sequence number, taxonomic diversity, metabarcode gaps, species age, or speciation rate (Rabosky et al. 2018). This challenges previous hypotheses (Polanco et al. 2021), and may instead reflect varying degrees of gene conservation among families as previously observed (Dziedzic et al. 2023). Overall, given the differences in over‐splitting and over‐merging error rates among orders (Figure 6) and the broad dispersion of intra‐taxa similarities (Figure 4) as previously noted in other taxonomic groups (Somervuo et al. 2017), the most robust approach for future studies would be to systematically reevaluate taxonomic resolution and metabarcode gaps for each study region's reference database.

Overall, although the likelihood of over‐splitting errors increases at higher similarity thresholds (Figure 2), the general tendency toward high over‐merging error rates following *de novo* clustering (Figure 3) supports the widespread use of the 99% threshold (Table S1). However, our *in silico* mock community analysis indicates that both metabarcode selection and threshold choice should be adjusted based on the intraspecific and interspecific diversity of the studied community. Specifically, diversity‐related biases shift from a higher risk of over‐splitting to a higher risk of over‐merging as species richness increases, with the rate of this transition depending on both the metabarcode and the similarity threshold used (Supplement S6). This balance between overestimating and underestimating diversity suggests that metabarcodes prone to over‐merging may be preferable for small communities, where the risk of over‐splitting is generally higher, while those prone to over‐splitting may be more appropriate for highly diverse communities (Figure 7). Although our *in silico* mock community analysis was conducted using the Neighbour‐Joining clustering method only since it was representative of the commonest pattern of error rate trends across *C*_*T*_ among clustering methods (Figure 3), further studies could help determine whether clustering method selection should also be adjusted based on community complexity as previously suggested (Chen et al. 2013; Wei et al. 2021).

The primary limitation of this study stems from reliance on the full mitogenome database of GenBank, which typically lacks the level of intraspecific variability present in traditional eDNA datasets. This may lead to a potential underestimation of over‐splitting risk. However, the construction of local whole‐mitogenome databases shows that the ratio of intra‐specific to inter‐specific variability is generally low for most families considered (Dziedzic et al. 2023). Another limitation is the exclusive focus on universal fish metabarcodes previously evaluated by Zhang et al. (2020), which excludes newly developed metabarcodes (e.g., NeoFish, 16S 200 and 16S 400; Hu et al. 2022; Milan et al. 2020), as well as the Am12S metabarcode (Evans et al. 2016) despite its poor in situ performance (Evans et al. 2017). Additionally, other COI metabarcodes that are not strictly fish‐specific (i.e., Leray‐XT, SeaDNA‐short an SeaDNA‐mid; Collins et al. 2019; Leray et al. 2013) were also excluded. In this study, we also opted to abbreviate the full primer names (both forward and reverse, see Table 1) following the convention established in the comprehensive evaluation by Zhang et al. (2020). Given the inconsistencies in metabarcode nomenclature across studies (e.g., Zhu and Iwasaki 2023), which complicate appropriate metabarcode selection, we strongly advocate for continued use of standardised metabarcode names in future eDNA studies. Finally, this study focuses solely on taxonomic resolution, whereas numerous other factors can influence biodiversity estimates, including environmental stability (i.e., metabarcode length), PCR efficiency (i.e., amplification, specificity, universality and reproducibility) and the completeness of reference database (Alberdi et al. 2018; Claver et al. 2022; Keck et al. 2023; Marques et al. 2021; Zhang et al. 2020). To date, primer performance has been evaluated in vivo against mock communities (Bylemans et al. 2018; Evans et al. 2016, 2017; Hänfling et al. 2016; Hilário et al. 2023; Schenekar et al. 2020; Shu et al. 2021), traditional sampling (Collins et al. 2019; Evans et al. 2017; Hu et al. 2022; Jackman et al. 2021; Shaw et al. 2016) or comparative analyses among primers (Bylemans et al. 2018; Claver et al. 2022; Kumar et al. 2022; Maiello et al. 2023; Polanco et al. 2021; Roblet et al. 2024; Zhang et al. 2020). These studies have collectively identified 12 different markers as the “best” option, depending on the methodology, criteria and set of primers/species tested, highlighting the lack of clear consensus. Some of the metabarcodes identified as having the highest taxonomic resolution in this study may still exhibit biases in vivo, including amplification efficiency issues (e.g., COI and CytB metabarcodes), specificity limitations (e.g., Fish16S) or reduced final resolution (e.g., MiFish). While the optimal approach would be to evaluate all available primers in vivo against mock communities with known DNA inputs per species, such studies used only 6 to 38 species. This underscores the importance of *in silico* evaluations as a complementary alternative.

In the future, multi‐marker approaches may offer the best solution for leveraging the advantages of different metabarcodes (Figure 7) despite their higher costs (Zhu and Iwasaki 2023). The recent online tool of Zhu and Iwasaki (2023), which focuses on Japanese marine environments, represents an initial step toward simplifying the selection of complementary metabarcodes, though it considers only over‐merging risk and universality without accounting for other biases (i.e., amplification efficiency, specificity and database completeness). New procedures integrating the geographic distribution of each taxon, the regional completeness of the reference database, and the diversification rate of lineages may also reduce errors whatever the metabarcode used (Polanco et al. 2025). Third‐generation sequencing now enables amplification‐free recovery of full mitogenomes from eDNA samples (Deiner et al. 2017; Ruiz et al. 2025), renewing interest in long markers like the highly resolutive FishF1‐R1 (Andújar et al. 2018), despite its potential specificity issues (Collins et al. 2019) and our finding that length does not necessarily provide greater taxonomic resolution. Ultimately, these methods could be further enhanced by mitochondrial enrichment techniques (e.g., differential centrifugation, exonuclease; Jo et al. 2019; Ramón‐Laca et al. 2023) and/or mitochondrial amplification strategies (e.g., RCA, long‐range PCR, targeted sequencing, hybridization capture; Dhorne‐Pollet et al. 2020; Emser et al. 2021; Li et al. 2023; Ramón‐Laca et al. 2023) if eDNA integrity is sufficiently preserved within mitochondria (Jo et al. 2021). If a multi‐marker approach is not feasible and additional evidence indicates that the FishF1‐R1 barcode is unsuitable for eDNA with third‐generation sequencing (e.g., persistence duration, amplification, specificity), the MiFish or Teleo02 metabarcodes represent the best option for communities with relatively low expected diversity. Conversely, if this is not the case, certain CytB metabarcodes (i.e., L14735c/c2, L14912 and L14841) may be preferred to further reduce the risk of over‐merging taxa (Figure 7).

## Author Contributions

E.R. and J.‐D.D. conceived the framework using BINs as a reference baseline. E.R. performed all bioinformatic analyses and created the new R package. J.‐D.D. manually aligned genes for a comparison with the automated metabarcode extraction developed by E.R.; T.L. reviewed codes developed by E.R. for the main analyses. D.M., T.L. and J.‐D.D. reviewed the manuscript written by E.R. and gave advice about figures.

## Conflicts of Interest

The authors declare no conflicts of interest.

## Supporting information


**Appendix S1:** men70069‐sup‐0001‐AppendixS1.docx.

## Data Availability

All data and scripts generated during this study are publicly accessible: https://doi.org/10.6084/m9.figshare.26014840. The most important R functions are provided with a manual to ease the evaluation of future fish metabarcodes and to apply it to other taxonomic groups: https://github.com/ruizeliot/eDNA_metabarcodes_resolution.
