# Supplementary material for: Benchmarking the Taxonomic Resolution of Fish eDNA Metabarcodes Against COI Barcodes
Source: Mol Ecol Resour. 2025 Oct 30;26(1):e70069. doi: 10.1111/1755-0998.70069 (PMC12627918; doi:10.1111/1755-0998.70069)
Supplement: Supplementary file 1 — Appendix S1: men70069‐sup‐0001‐AppendixS1.docx. [file MEN-26-e70069-s001.docx]

**Supplementary material**

**Benchmarking the taxonomic resolution of fish eDNA metabarcodes against COI barcodes**

**Authors:** Eliot Ruiz ^1^, Thomas Lamy ^1^, David Mouillot ^1^, Jean-Dominique Durand ^1^

^1^ MARBEC, University of Montpellier, IRD, IFREMER, CNRS, Montpellier, France

**Contact information:** [eliotruiz3@gmail.com](mailto:eliot.ruiz@gmail.com)

**SUPPLEMENT 1 – METABARCODE EXTRACTION PROCESS**


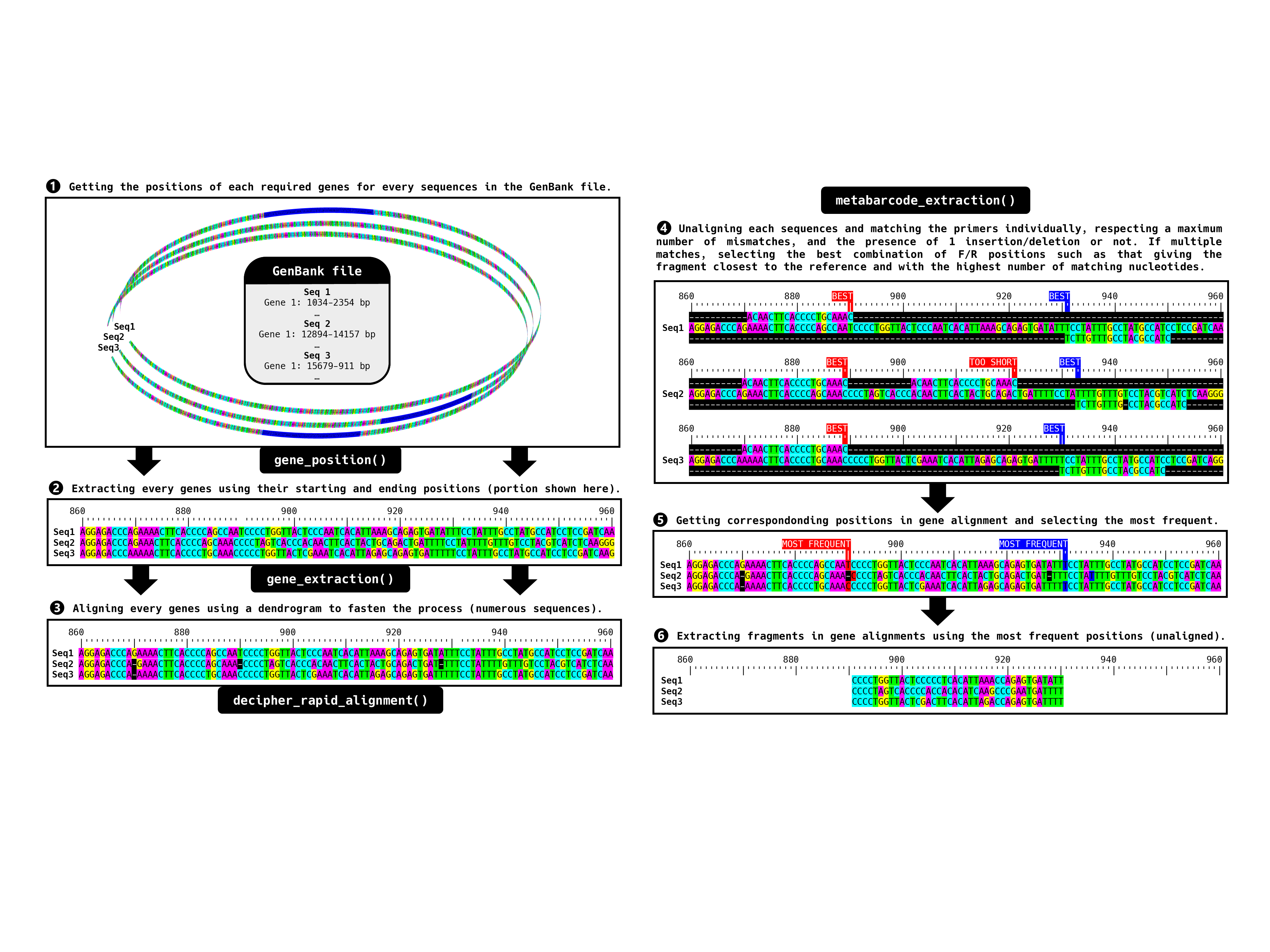


FIGURE S1 | Schematic representation of the metabarcode extraction process from whole NCBI mitogenomes implemented in the new “eDNAevaluation” R package. First, gene starting and ending positions are retrieved from the GenBank file using the function *gene_position()*. Then, genes are extracted using *gene_extraction()* and subsequently aligned in a computationally efficient way using *decipher_rapid_aligment()*. Finally, each primer set provided is used by the function *metabarcode_extraction()* to select the most likely starting and ending position of metabarcodes in the alignment and further proceed to their extraction.

**SUPPLEMENT 2 – INTRA-BIN AND INTER-BIN ANALYSES**


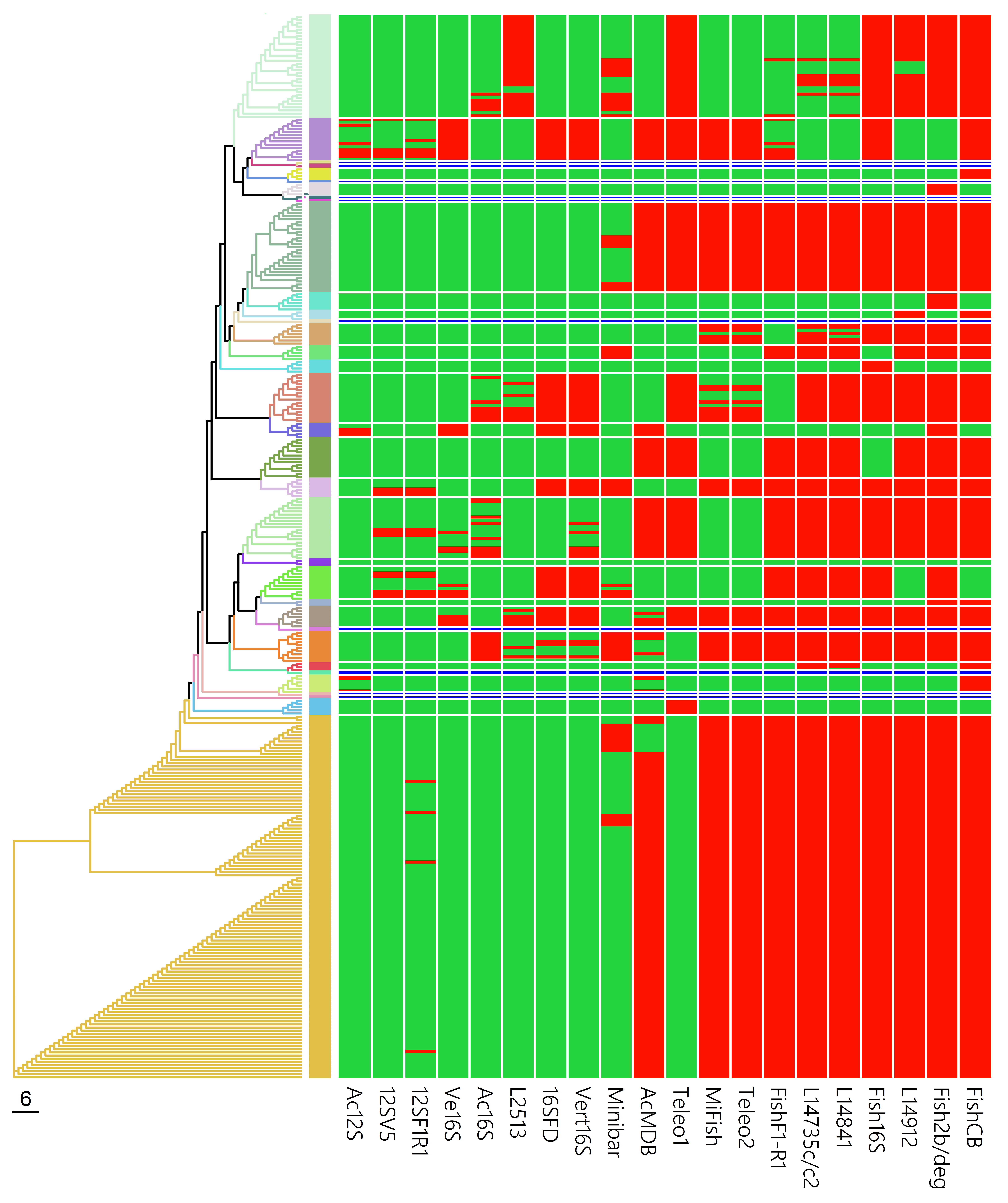


FIGURE S2 | Visual representation of over-splitting errors at 99% (most used similarity threshold in metabarcoding) occurring in a small subset: the Salmonidae family containing 11 genera, 34 BIN, and 342 complete mitogenomes in our dataset. The Neighbour-Joining cladogram (*i.e.*, unscaled branches) on the left was constructed based on the FishF1-R1 barcode, using a K80 distance. Each BIN is identified by a unique color in the tree, and corresponds to a block delineated with white lines on the right. For every 20 metabarcodes, sequences wrongly considered different from others (*S_XY_ < S_T_*) while belonging to the same BIN are represented in red (“intra-BIN errors”). Conversely, sequences within a BIN with *S_XY_* *≥ S_T_* are represented in green. BINs containing only one sequence were excluded from our analysis and are represented as blue blocks. Metabarcodes were ordered from left to right by increasing number of intra-BIN errors for the Salmonidae family.


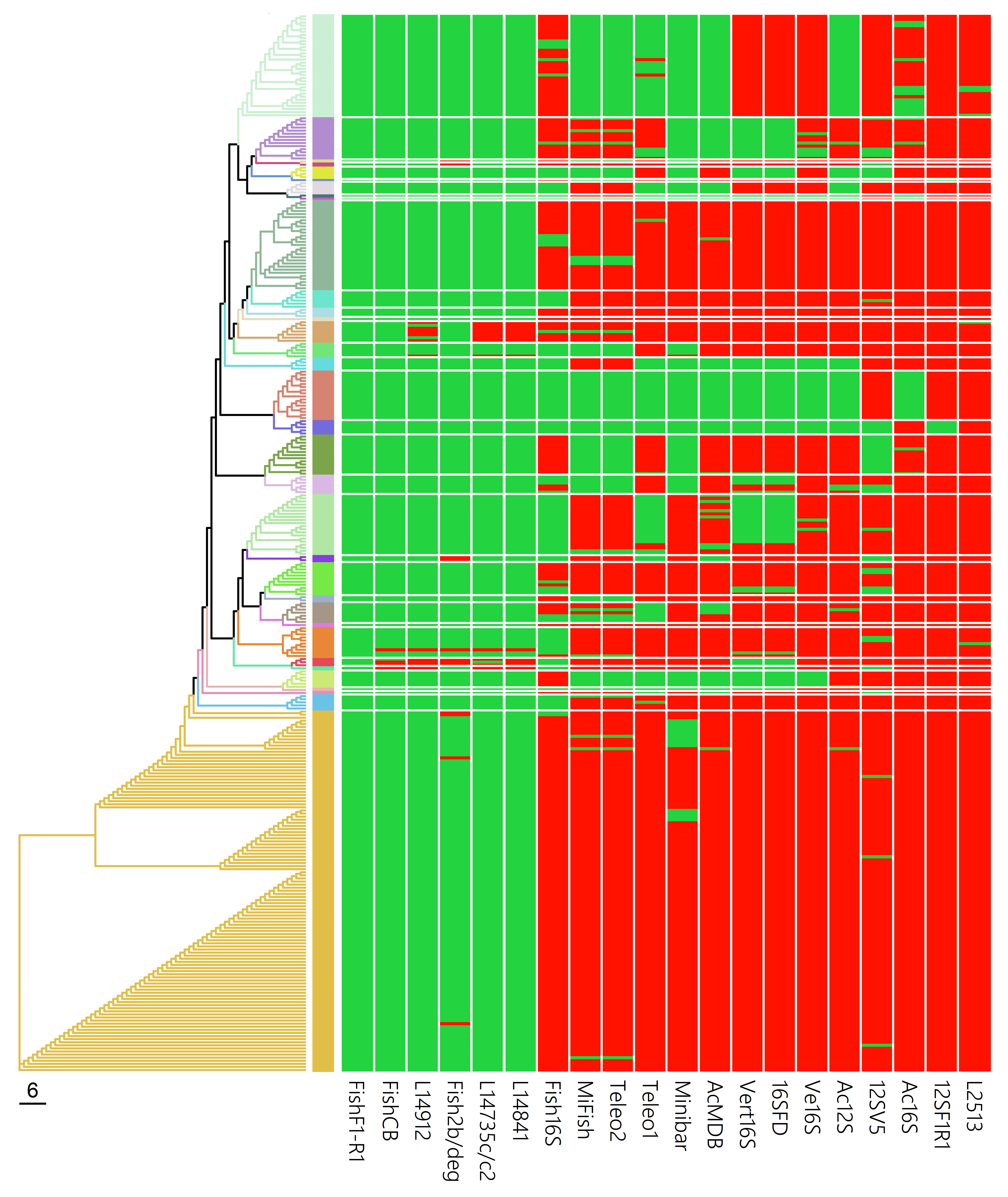


FIGURE S3 | Visual representation of over-merging errors at 99% (most used similarity threshold in metabarcoding) occurring in a small subset: the Salmonidae family containing 11 genera, 34 BIN, and 342 complete mitogenomes in our dataset. The Neighbour Joining phylogenetic tree on the left was created using the FishF1-R1 barcode. Each BIN is identified by a unique color in the tree and corresponds to a block delineated with white lines on the right. For every 20 (meta)barcodes, sequences belonging to two different BINs but wrongly grouped together (*S_XY_ ≥ S_T_*) are represented in red (“inter-BIN errors”). Conversely, sequences from different BIN accurately discriminated (*S_XY_ < S_T_*) are represented in green. Metabarcodes were ordered from left to right by increasing number of inter-BIN errors for the Salmonidae family.

v


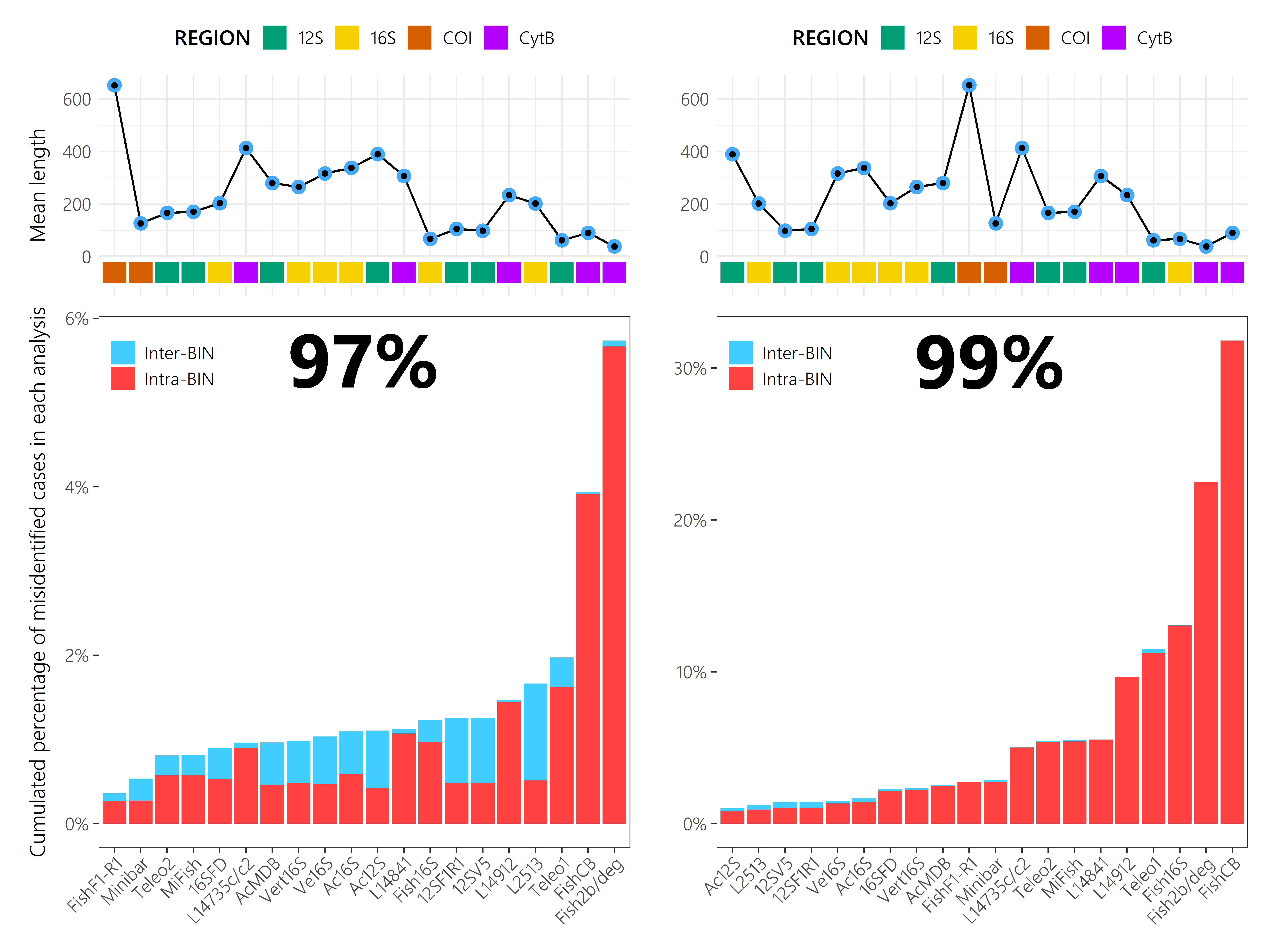


FIGURE S4 | Stacked proportions of intra-BIN (over-splitting; red) and inter-BIN (over-merging; blue) errors in the intra-BIN and inter-BIN analyses using the 97% similarity threshold commonly used in barcoding (left plot), and the 99% *S_T_* commonly used in metabarcoding (right plot). Metabarcodes are ordered from left to right by increasing cumulated proportion of intra-BIN and inter-BIN errors. The average length after extraction is represented on top of each metabarcode as a blue point, along with the mitochondrial region on which the metabarcode is located.


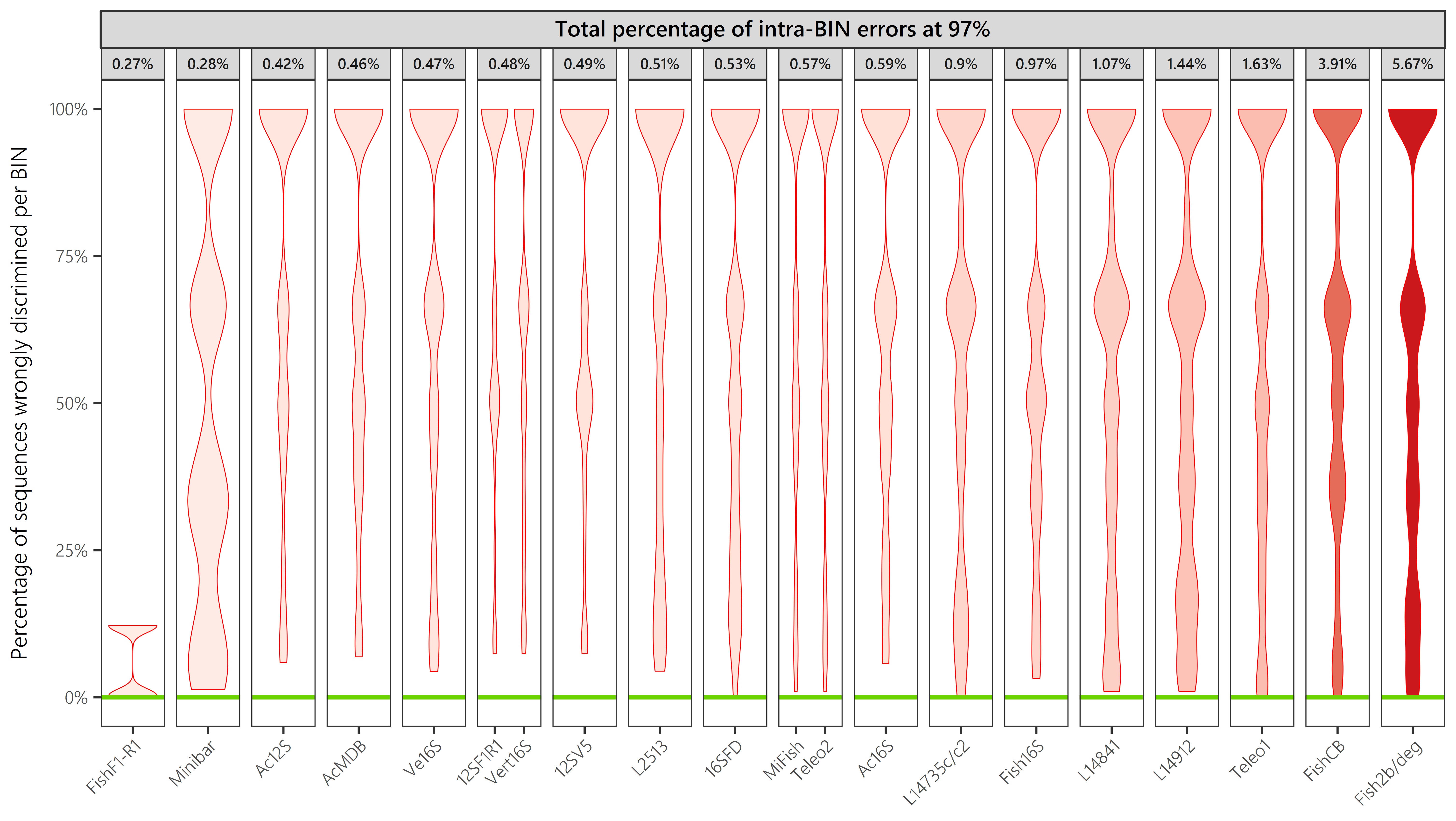


FIGURE S5 | Violin plot representing the proportion of intra-BIN errors (over-splitting) per BIN with a similarity threshold of 97% commonly used in barcoding, excluding BIN for which no sequences were wrongly considered different (green line at 0%). The intensity of violins’ colors is proportional to the global percentage of intra-BIN errors. Metabarcodes are also grouped by similar intra-BIN error rates indicated on top of each panel.


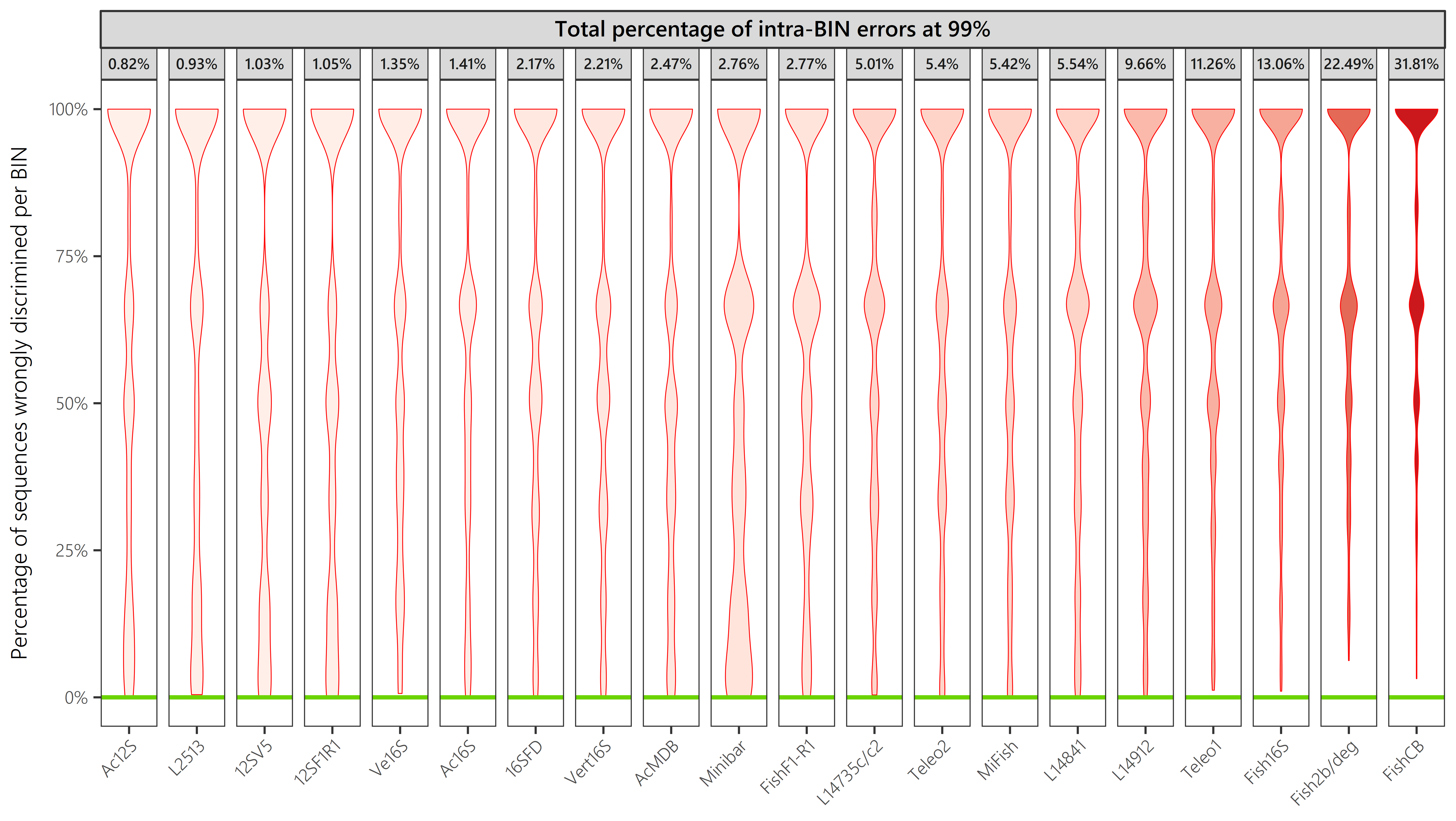


FIGURE S6 | Violin plot representing the proportion of intra-BIN errors (over-merging) per BIN with a similarity threshold of 99% commonly used in metabarcoding, excluding BIN for which no sequences were wrongly considered different (green line at 0%). The intensity of violins’ colors is proportional to the global percentage of intra-BIN errors. Metabarcodes are also grouped by similar intra-BIN error rates indicated on top of each panel.

**SUPPLEMENT 3 – EFFECT OF CLUSTERING THRESHOLDS AND METHODS**

**
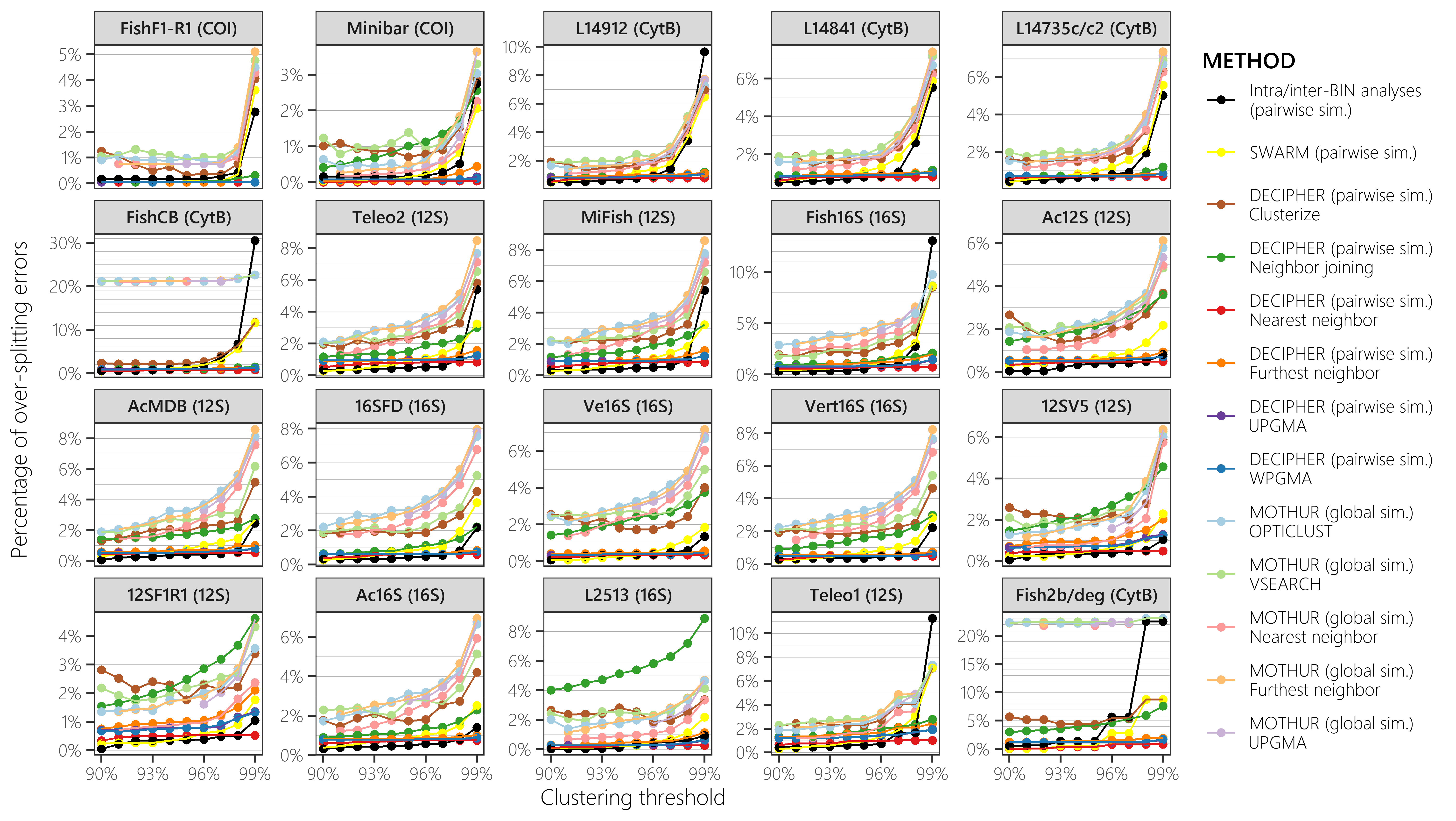
**

FIGURE S7 | Cumulated proportions of over-splitting errors in the clusters created with various methods implemented in MOTUR, SWARM, and DECIPHER as a function of *C_T_*, along with results presented in Figure 2 established from intra/inter-BIN analyses. Metabarcodes are ordered as in Figure 2, according to their best score in the error assessment, and the mitochondrial gene targeted by the primer set is provided within parentheses.

.


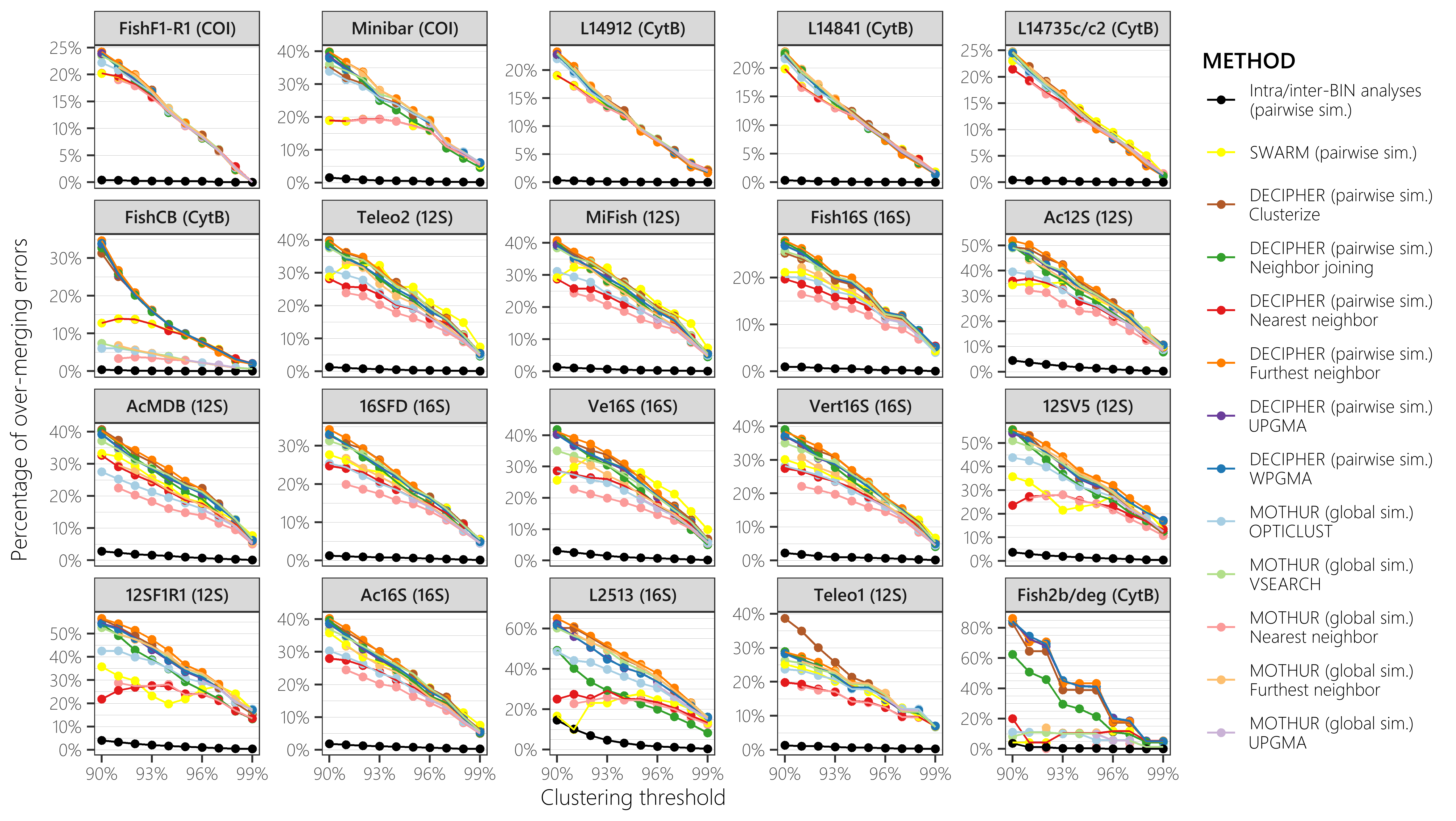


FIGURE S8 | Cumulated proportions of over-merging errors in the clusters created with various methods implemented in MOTUR, SWARM, and DECIPHER as a function of *C_T_*, along with results presented in Figure 2 established from intra/inter-BIN analyses. Metabarcodes are ordered as in Figure 2, according to their best score in the error assessment, and the mitochondrial gene targeted by the primer set is provided within parentheses.

.

| **PRIMERS** | **GENE** | **PROGRAM** | **TYPE** | **METHOD** | ***C_OPT_*** | **OVER-SPLITTING** | **OVER-MERGING** | **SUM** |
| --- | --- | --- | --- | --- | --- | --- | --- | --- |
| FishF1-R1 | COI | DECIPHER | Pairwise | Furthest neighbor, UPGMA & WPGMA | 99% | 0.04% | 0% | 0.04% |
| L14735c/c2 | CytB |  |  |  | 99% | 0.83% | 1.17% | 1.99% |
| L14841 | CytB |  |  | Furthest neighbor | 99% | 1.05% | 1.35% | 2.4% |
| L14912 | CytB |  |  |  | 99% | 1.13% | 1.7% | 2.82% |
| FishCB | CytB |  |  | Nearest neighbor | 99% | 0.79% | 2.1% | 2.88% |
| Fish2b/deg | CytB |  |  |  | 91/92% | 0.08% | 4.23% | 4.3% |
| Vert16S | 16S |  |  | Furthest neighbor | 99% | 0.71% | 4.63% | 5.35% |
| Minibar | COI |  |  | Nearest neighbor | 99% | 0.04% | 5.47% | 5.51% |
| 16SFD | 16S |  |  | Furthest neighbor | 99% | 0.83% | 4.84% | 5.66% |
| Fish16S | 16S |  |  | Nearest neighbor | 99% | 0.71% | 5.4% | 6.11% |
| Ac16S | 16S |  |  | Furthest neighbor | 99% | 1.01% | 5.24% | 6.25% |
| MiFish | 12S |  |  | Nearest neighbor | 99% | 0.83% | 5.48% | 6.3% |
| Teleo2 | 12S |  |  |  | 99% | 0.83% | 5.52% | 6.35% |
| Ve16S | 16S |  |  | Furthest neighbor | 99% | 0.56% | 5.85% | 6.41% |
| AcMDB | 12S |  |  |  | 99% | 1.01% | 5.83% | 6.84% |
| Teleo1 | 12S |  |  | Nearest neighbor | 99% | 1.01% | 6.97% | 7.98% |
| L2513 | 16S | SWARM |  | SWARM | 91% | 0.11% | 10% | 10.11% |
| Ac12S | 12S | DECIPHER |  | Nearest neighbor | 99% | 0.49% | 9.74% | 10.22% |
| 12SV5 | 12S |  |  |  | 99% | 0.49% | 13.42% | 13.9% |
| 12SF1R1 | 12S |  |  |  | 99% | 0.53% | 13.52% | 14.04% |

TABLE S1 | Clustering methods producing the smallest cumulated percentage of over-splitting and over-merging errors per metabarcode. Optimal similarity thresholds (*S_OPT_*) and associated minimal error rates with intra/inter-BIN analyses based on pairwise similarity are presented in Figure 2. Optimal clustering thresholds (*C_OPT_*) per clustering method and per metabarcode are presented in Figure 3.

**SUPPLEMENT 4 – METABARCODE GAPS**


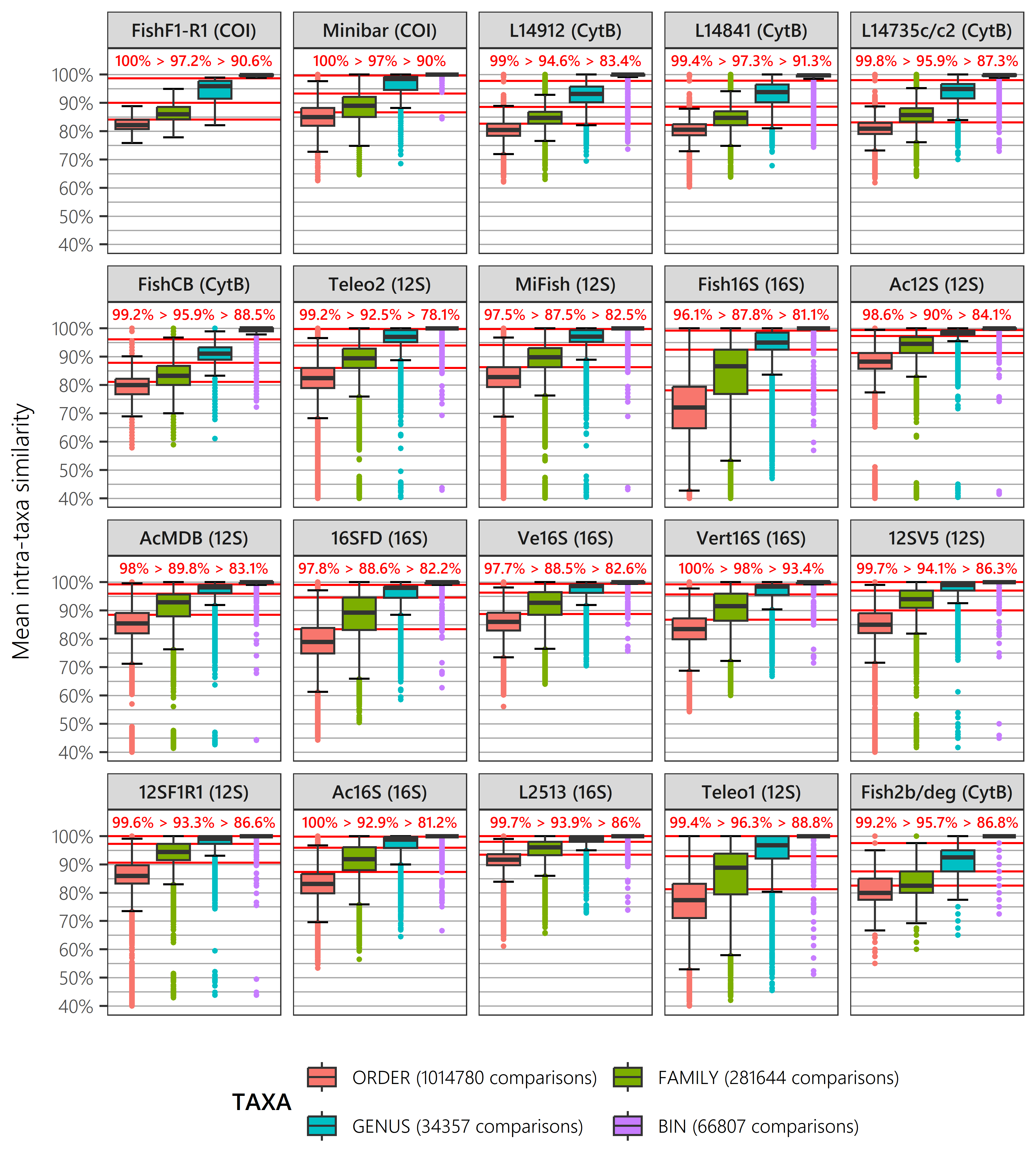


FIGURE S9 | Distribution of the intra-taxa pairwise similarity (*S_XY_*) for all comparisons per taxonomic level (indicated in the legend) and per metabarcode. Similarity thresholds between taxonomic levels represented as red numbers and horizontal lines were computed following the method described in Figure 4. However, it must be noted that they are biased toward taxa containing large numbers of subtaxa and/or sequences in our dataset since it greatly increases the number of pairwise comparisons that overwhelm other taxa with fewer comparisons. Metabarcodes are ordered as in Figure 2, according to their best score in the error assessment.

**SUPPLEMENT 4 – INTRA-ORDER VARIABILITY OF TAXONOMIC RESOLUTION**


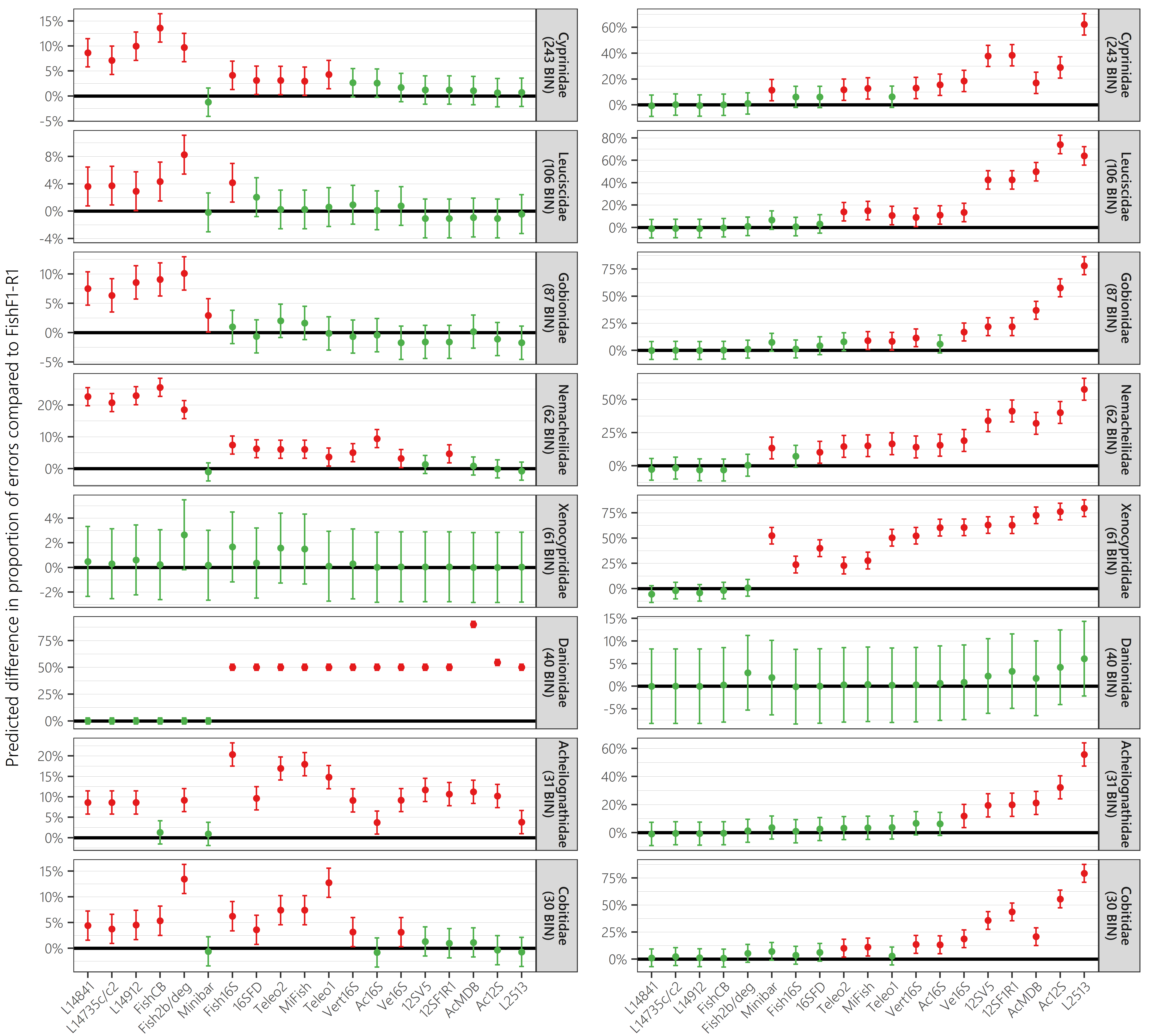


FIGURE S10 | The difference in predicted over-splitting errors (left column) and over-merging error rates (right column) relative to the FishR1-R1 barcode (bold black line) for each of the 19 metabarcodes and the eight most prevalent Cypriniformes families of our database (number of BINs > 30). A positive percentage indicates that the metabarcode exhibits a higher error rate than FishF1-R1 (black bold line centered on 0%) for the same order, while a negative percentage indicates a lower error rate. Red coloration denotes a statistically significant difference from 0. Green coloration indicates a non-significant difference. The 95% Wald confidence interval is represented as an error bar around each prediction. Metabarcodes are arranged from left to right in order of increasing total predicted over-splitting and over-merging errors.

**SUPPLEMENT 6 – *IN SILICO* MOCK COMMUNITIES**

To assess the impact of metabarcodes’ taxonomic errors on biodiversity estimate, we conducted an *in-silico* simulation of the sequencing output that could be obtained from a diverse array of mock communities (*i.e.*, communities with known composition) using the custom function *perception_mock_communities*. The mock communities varied in their number of unique sequences (*X_SEQ_*) from 10 to 1,000 by increments of 10, reflecting the typical range of observed fish richness detected in aquatic eDNA samples (*e.g.*, Yamamoto et al., 2017; Mathon et al. 2023). Additionally, communities’ diversity expressed in terms of BINs (*Y_BIN_*), was randomly sampled from the uniform distribution between [0.5 × *X_SEQ_*; 2 × *X_SEQ_*], with 1,000 iterations per *X_SEQ_* value (**Figure S10**). To enhance biological realism (*e.g.,* accounting for eDNA degradation and PCR biases), we assumed that only a fraction of *X_BIN_* could be successfully sequenced (*Y_BIN_*), and that the number of unique eDNA sequences within each BIN was randomly sampled to ensure that the total equals *Y_SEQ_*. The approach prevented random selection from the final dataset of 5,438 sequences, which would lead to underestimating over-splitting error rates, given that the probability of randomly sampling sequences belonging to the same BIN is low (0.45%), whereas this probability is much higher in eDNA samples (*e.g.*, Yamamoto et al., 2017). By simulating 100,000 random communities with varying overall diversity (*Y_BIN_*) and total unique sequences (*Y_SEQ_*), we aimed to capture the broad range of potential over-splitting and over-merging error values (**Figure S10**). We applied the custom function *clusters_per_primers_decipher* to create a Neighbor Joining dendrogram per metabarcode and further retrieve OTUs for all *S_T_* between 90% and 99%. Finally, for each of the 100,000 communities, we computed the number of OTUs detected by each metabarcode (*Z_OTU_*) at each *S_T_*. A ratio *Z_OTU_* /*Y_BIN_* > 1 indicated an overestimation of diversity compared to the BIN baseline, whereas *Z_OTU_* /*Y_BIN_* < 1 indicated an underestimation (**Figure S10**).

Using the Neighbor Joining method which is representative of most clustering techniques (**Figure 3**), we demonstrated that the mean number of OTUs detected consistently exceeded the actual number of BINs when the number of eDNA sequences was small, though variability remained high. However, the predominance of over-splitting errors, which contributes to this biodiversity overestimation, was gradually offset by an increased probability of over-merging errors as eDNA sequence pools became larger and more diverse. Overall, the biodiversity bias exhibited a near-linear decline with *Y_SEQ_* (mean *R*² = 0.98 for all *S_T_* and metabarcodes). However, diversity underestimation (*e.g.,* max = 23.2%) was generally more pronounced than diversity overestimation (*e.g.,* max = 2.6%), as the tipping point typically occurs at low *Y_SEQ_*, particularly for *S_T_* below 98% (**Figures S11-S20**). Metabarcodes previously identified as more sensitive to over-splitting errors, such as L2513 (16S), 12SV5 (12S), and 12SF1R1 (12S), exhibited the steepest slopes. Conversely, flatter slopes were observed for CytB metabarcodes and the COI barcode FishF1-R1, which were less affected by over-merging errors at *S_T_* = 99% (**Figure 2**), mitigating the increased likelihood of over-splitting errors at high *Y_SEQ_* (**Figure 5**).

Overall, our simulations, in which *Y_SEQ_* closely approximated *Y_BIN_* at high *Y_SEQ_* indicated that the optimal similarity threshold for minimizing biodiversity bias |*Z_OTU_*/*Y_BIN_*| in eDNA sequences pools of unknown diversity is 99% for all metabarcodes as previously observed (**Figure 3**). However, exceptions were noted for CytB metabarcodes, where the optimal *S_T_* values were as follows: FishCB (90%), L14841 & L14912 (96%), Fish2b/2deg (97%), L14735c/c2 (98%). Among these, L14735c/c2 exhibited the lowest biodiversity bias in our simulations (0.30%), making it the most reliable CytB metabarcode at its optimal threshold. For other mitochondrial genes, the metabarcodes that minimized biodiversity bias at *S_T_* = 99% were MiFish/Teleo2 (12S, 0.66%), 16SFD (16S, 0.43%), and FishF1-R1 (COI, 0.24%). These findings suggest that while a 99% similarity threshold generally provides the best balance between over-splitting and over-merging errors, gene-specific adjustments may be necessary for optimal biodiversity estimation.


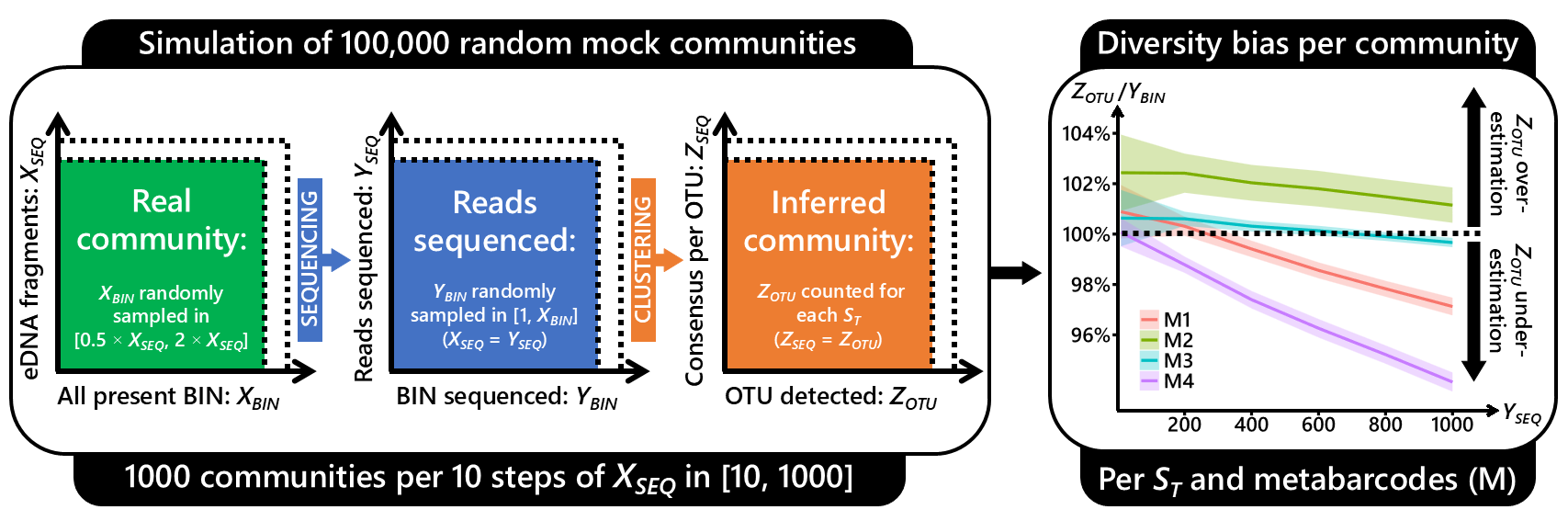


FIGURE S111 | Schematic representation of the framework used for the in silico mock community analysis. To evaluate whether a given metabarcode tends to overestimate or underestimate diversity depending on community composition, we simulated randomly in silico mock communities of increasing size (*Y_SEQ_*) and diversity (*Y_BIN_*) based on sequencing a natural community with a varying BIN redundancy (*X_BIN_*). Using a single Neighbor Joining clustering method, we quantified the number of OTUs detected (*Z_OTU_*) for each S_T_ and metabarcode, comparing them to the actual number of BINs sequenced (*Y_BIN_*) to assess how diversity evolves with increasing *Y_SEQ_*.


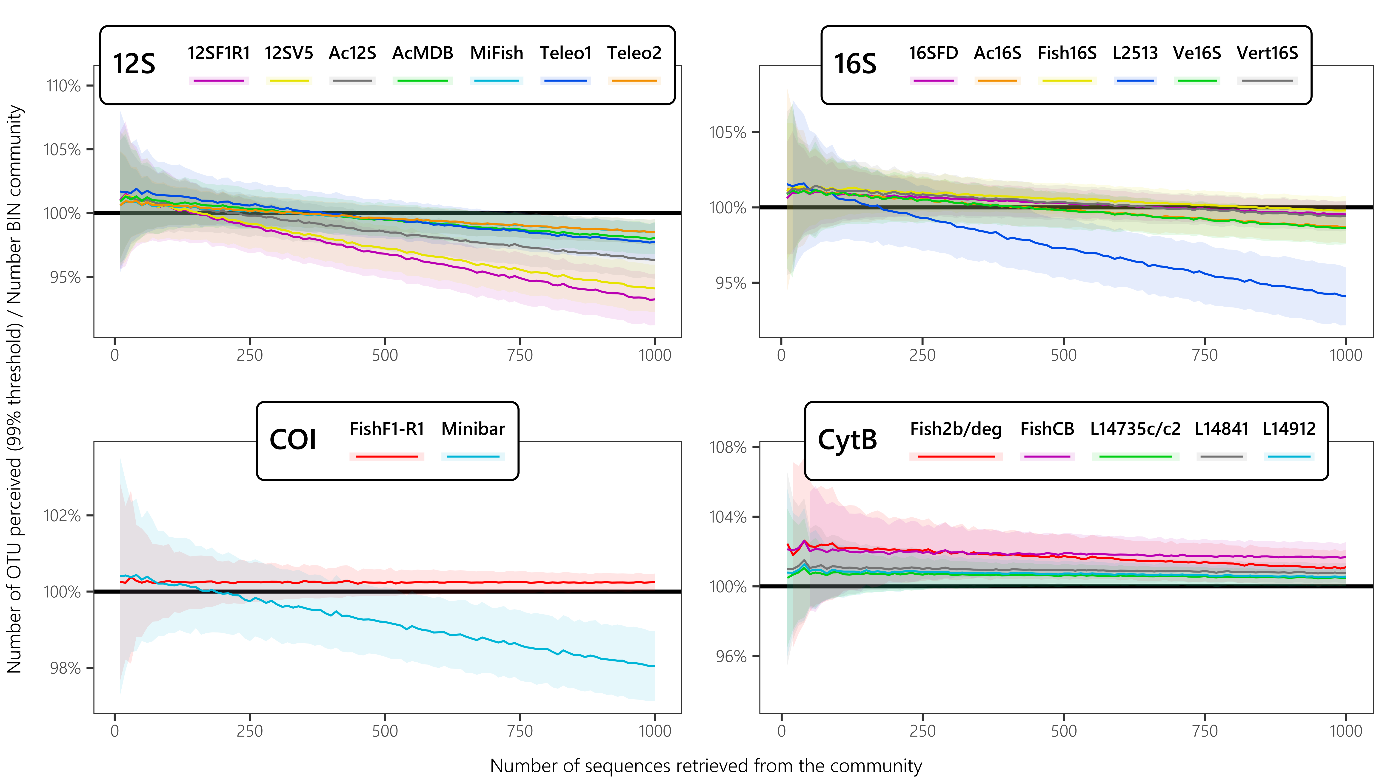


FIGURE S12 | Impact of taxonomic resolution variability on metabarcode-based biodiversity assessment. The number of distinct OTUs (*Z_OTU_*) was determined using a Neighbor Joining clustering algorithm with a 99% similarity threshold. This analysis was performed for 100,000 eDNA sequences, each with known size and biodiversity, which were retrieved from in silico mock communities with defined BIN composition (Figure S11). The biodiversity bias, defined as the overestimation or underestimation of the number of BINs, was calculated as the ratio between the number of OTUs (*Z_OTU_*) generated and the actual number of BINs (*Y_BIN_*). Solid colored lines represent the mean biodiversity bias across all 1,000 communities *Z_SEQ_* increments, while the shaded area indicates the standard deviation.


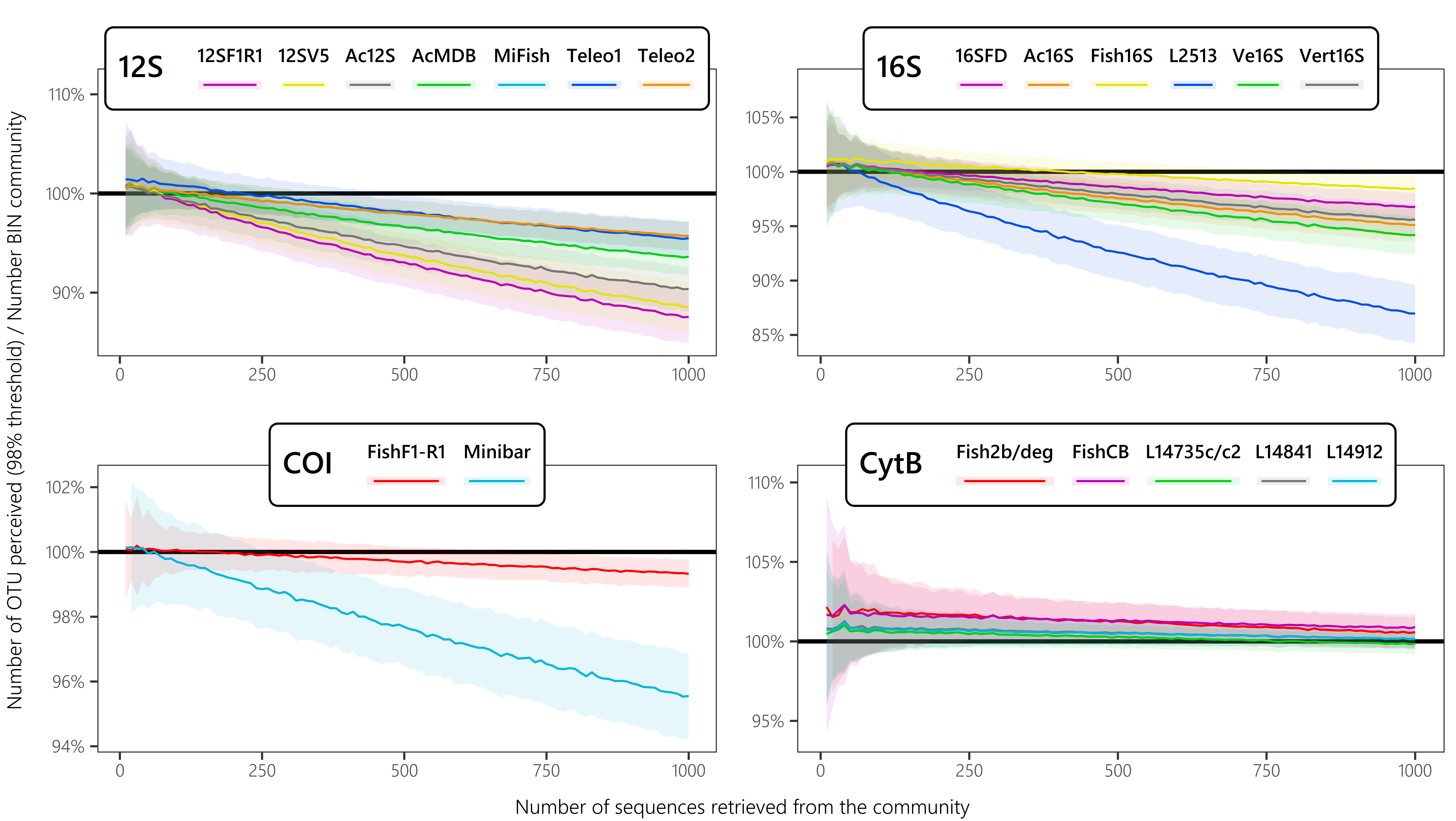

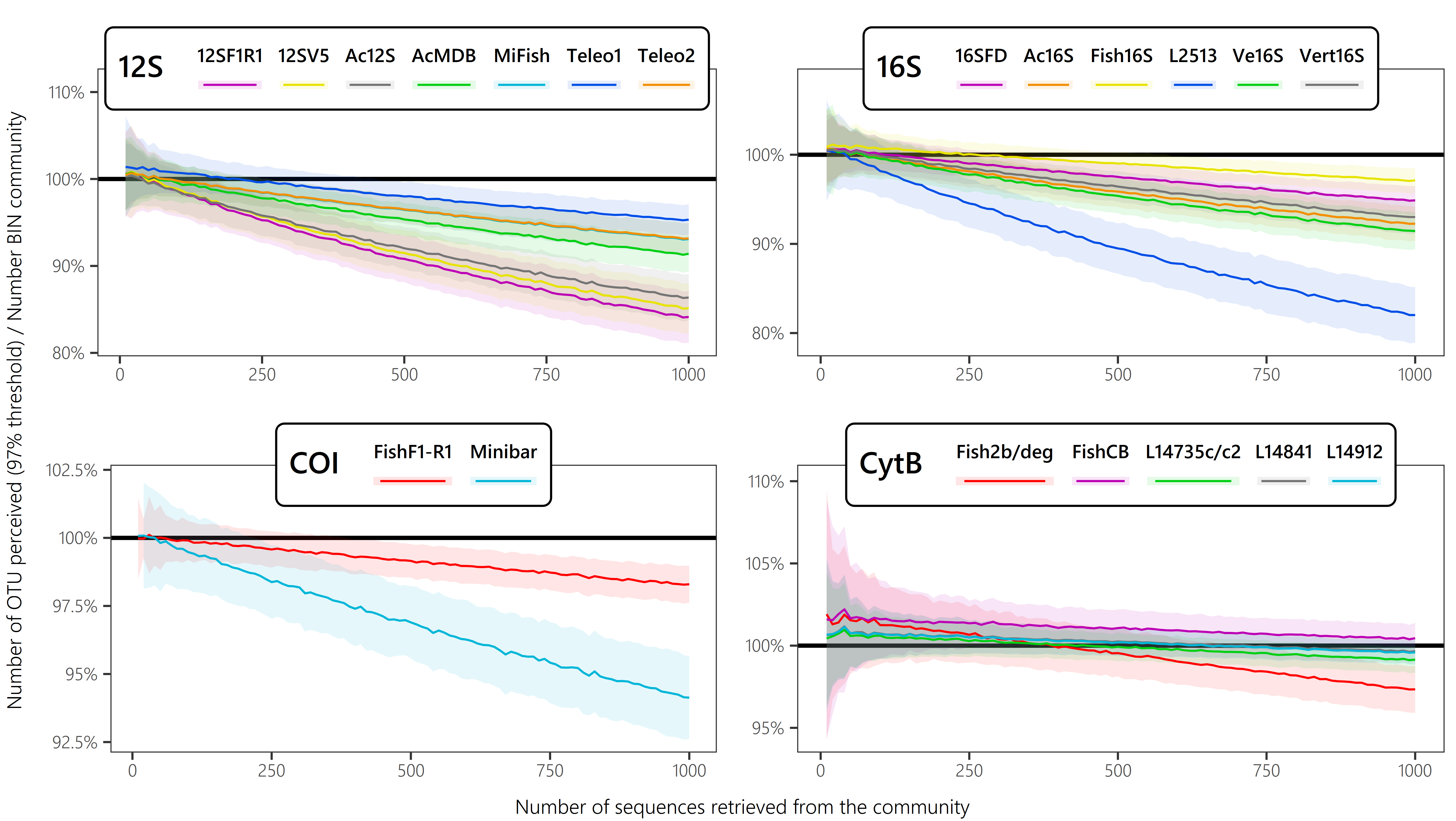


FIGURE S13 | Taxonomic resolution assessment using *in silico* mock communities and a 98% similarity threshold. Each line represents the mean ratio between the number of OTU (*Z_OTU_*) perceived and the actual number of BIN (*Y_BIN_*) across the 1,000 iterations per number of eDNA sequences (*Y_SEQ_*) interval, with its standard deviation in shaded color around (see Figure S11 for details about this analysis). Values higher than 100% correspond to an overestimation of diversity and conversely for values lower than 100%.

FIGURE S14 | Taxonomic resolution assessment using *in silico* mock communities and a 97% similarity threshold. Each line represents the mean ratio between the number of OTU (*Z_OTU_*) perceived and the actual number of BIN (*Y_BIN_*) across the 1,000 iterations per number of eDNA sequences (*Y_SEQ_*) interval, with its standard deviation in shaded color around (see Figure S11 for details about this analysis). Values higher than 100% correspond to an overestimation of diversity and conversely for values lower than 100%.


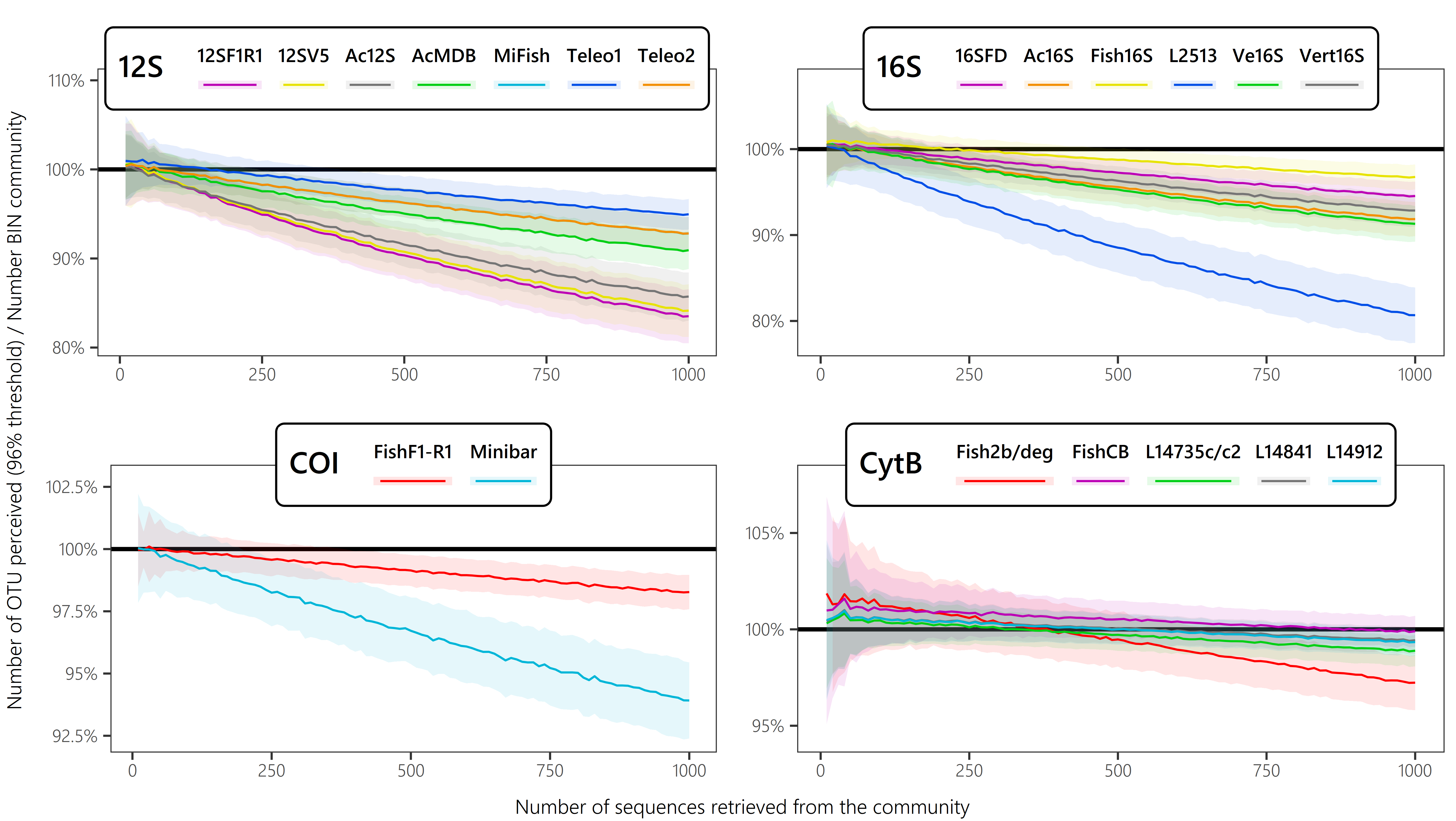


FIGURE S15 | Taxonomic resolution assessment using *in silico* mock communities and a 96% similarity threshold. Each line represents the mean ratio between the number of OTU (*Z_OTU_*) perceived and the actual number of BIN (*Y_BIN_*) across the 1,000 iterations per number of eDNA sequences (*Y_SEQ_*) interval, with its standard deviation in shaded color around (see Figure S11 for details about this analysis). Values higher than 100% correspond to an overestimation of diversity and conversely for values lower than 100%.


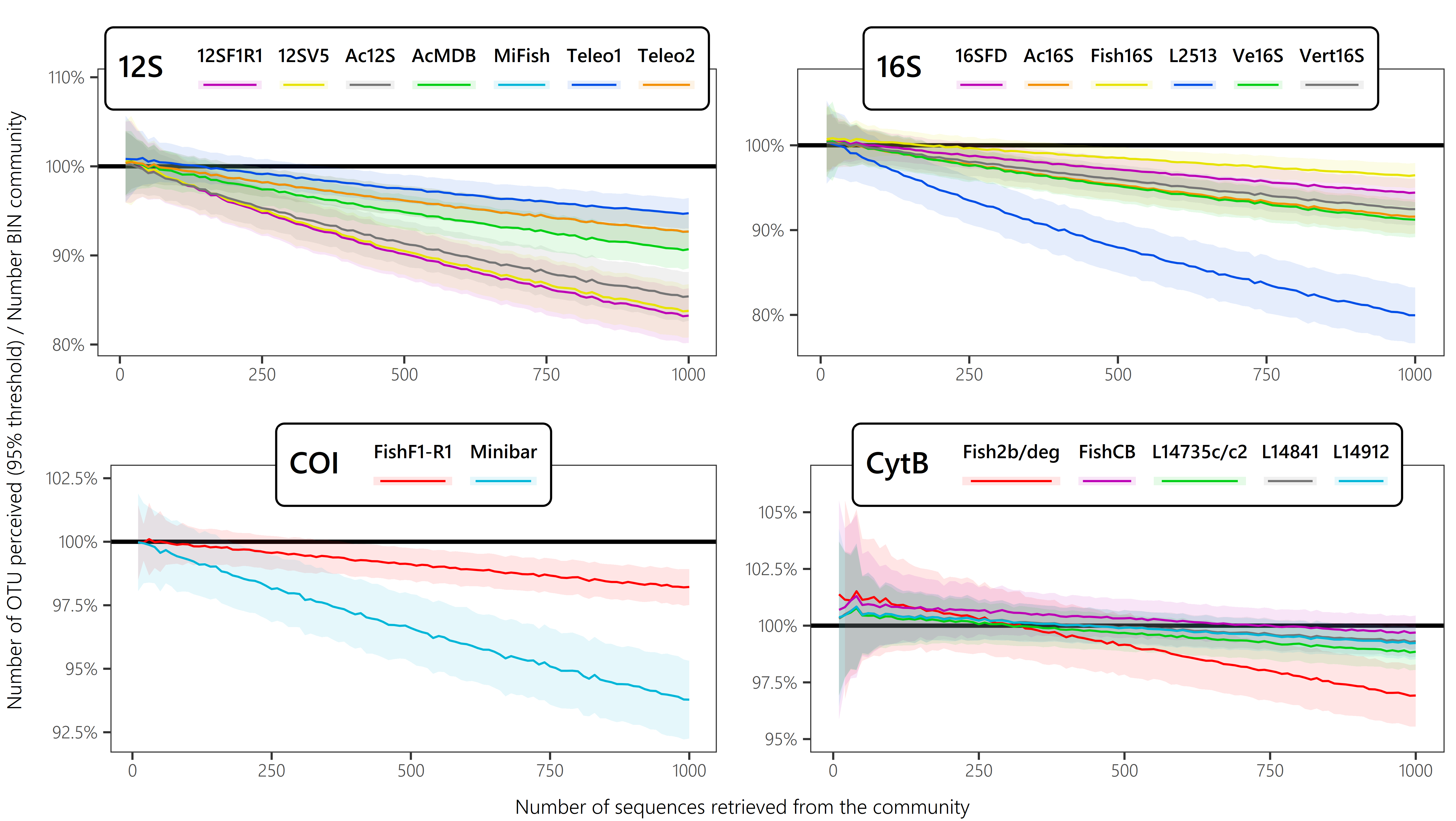


FIGURE S16 | Taxonomic resolution assessment using *in silico* mock communities and a 95% similarity threshold. Each line represents the mean ratio between the number of OTU (*Z_OTU_*) perceived and the actual number of BIN (*Y_BIN_*) across the 1,000 iterations per number of eDNA sequences (*Y_SEQ_*) interval, with its standard deviation in shaded color around (see Figure S11 for details about this analysis). Values higher than 100% correspond to an overestimation of diversity and conversely for values lower than 100%.


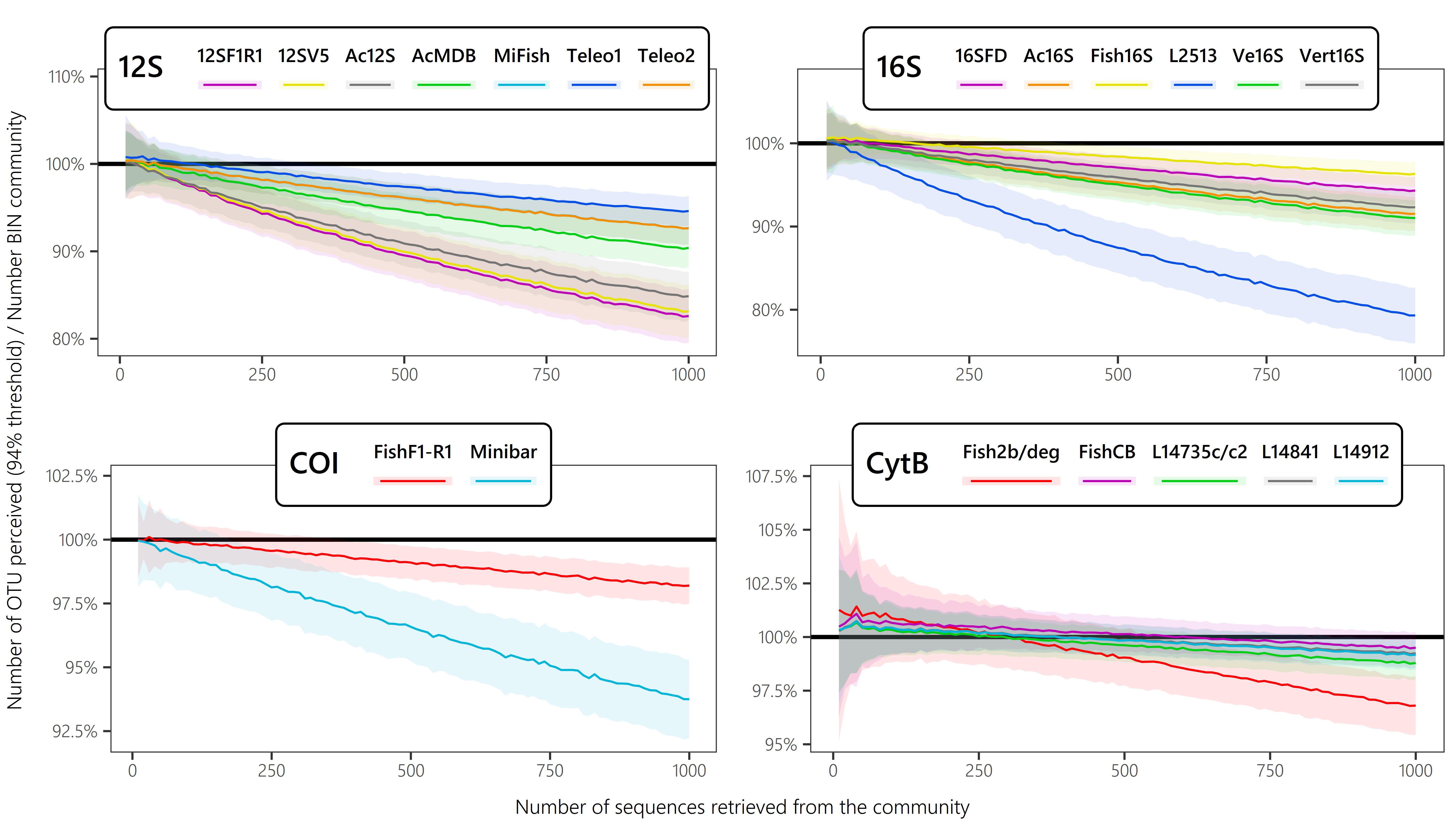


FIGURE S17 | Taxonomic resolution assessment using *in silico* mock communities and a 94% similarity threshold. Each line represents the mean ratio between the number of OTU (*Z_OTU_*) perceived and the actual number of BIN (*Y_BIN_*) across the 1,000 iterations per number of eDNA sequences (*Y_SEQ_*) interval, with its standard deviation in shaded color around (see Figure S11 for details about this analysis). Values higher than 100% correspond to an overestimation of diversity and conversely for values lower than 100%.


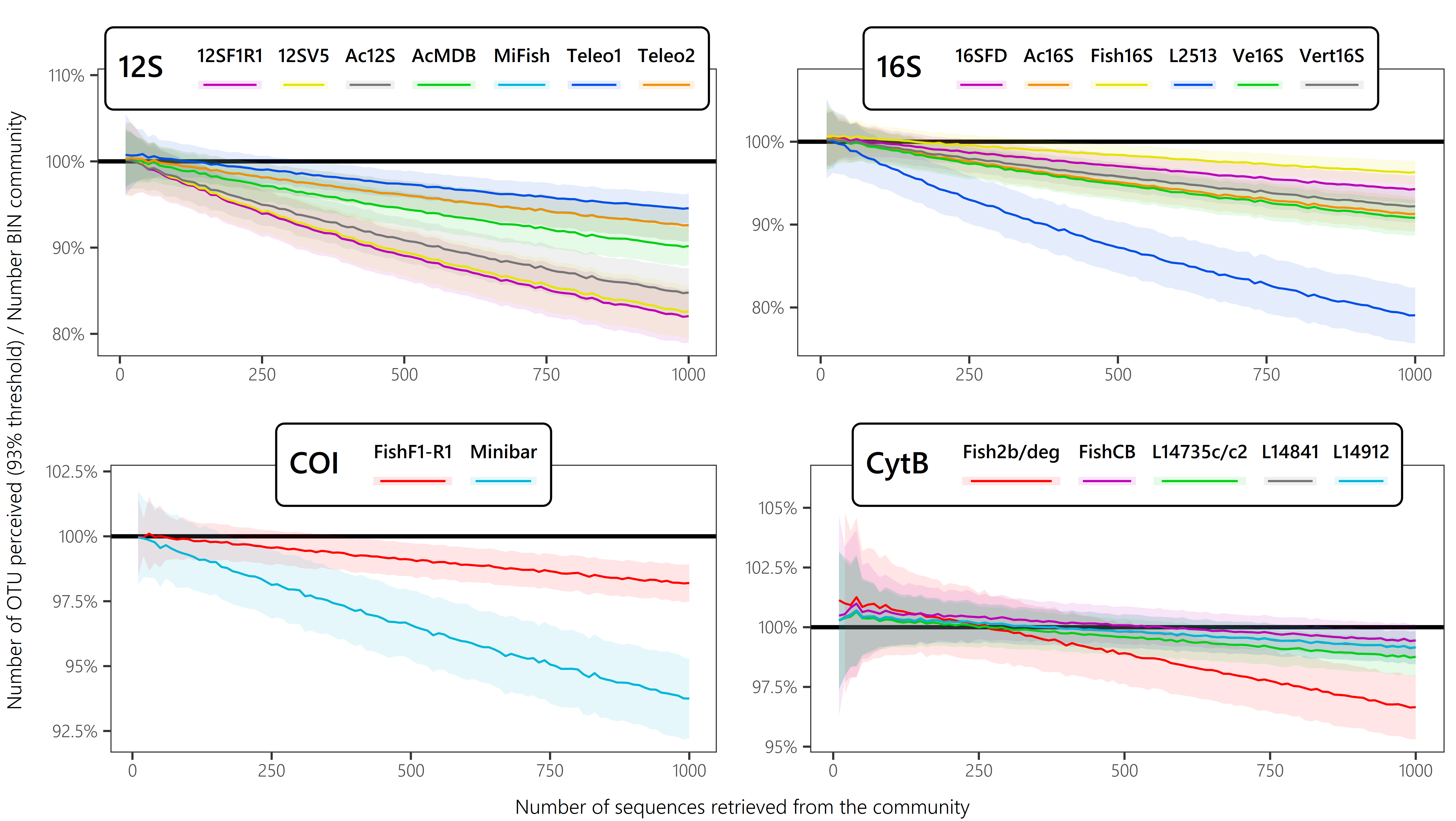


FIGURE S18 | Taxonomic resolution assessment using *in silico* mock communities and a 93% similarity threshold. Each line represents the mean ratio between the number of OTU (*Z_OTU_*) perceived and the actual number of BIN (*Y_BIN_*) across the 1,000 iterations per number of eDNA sequences (*Y_SEQ_*) interval, with its standard deviation in shaded color around (see Figure S11 for details about this analysis). Values higher than 100% correspond to an overestimation of diversity and conversely for values lower than 100%.


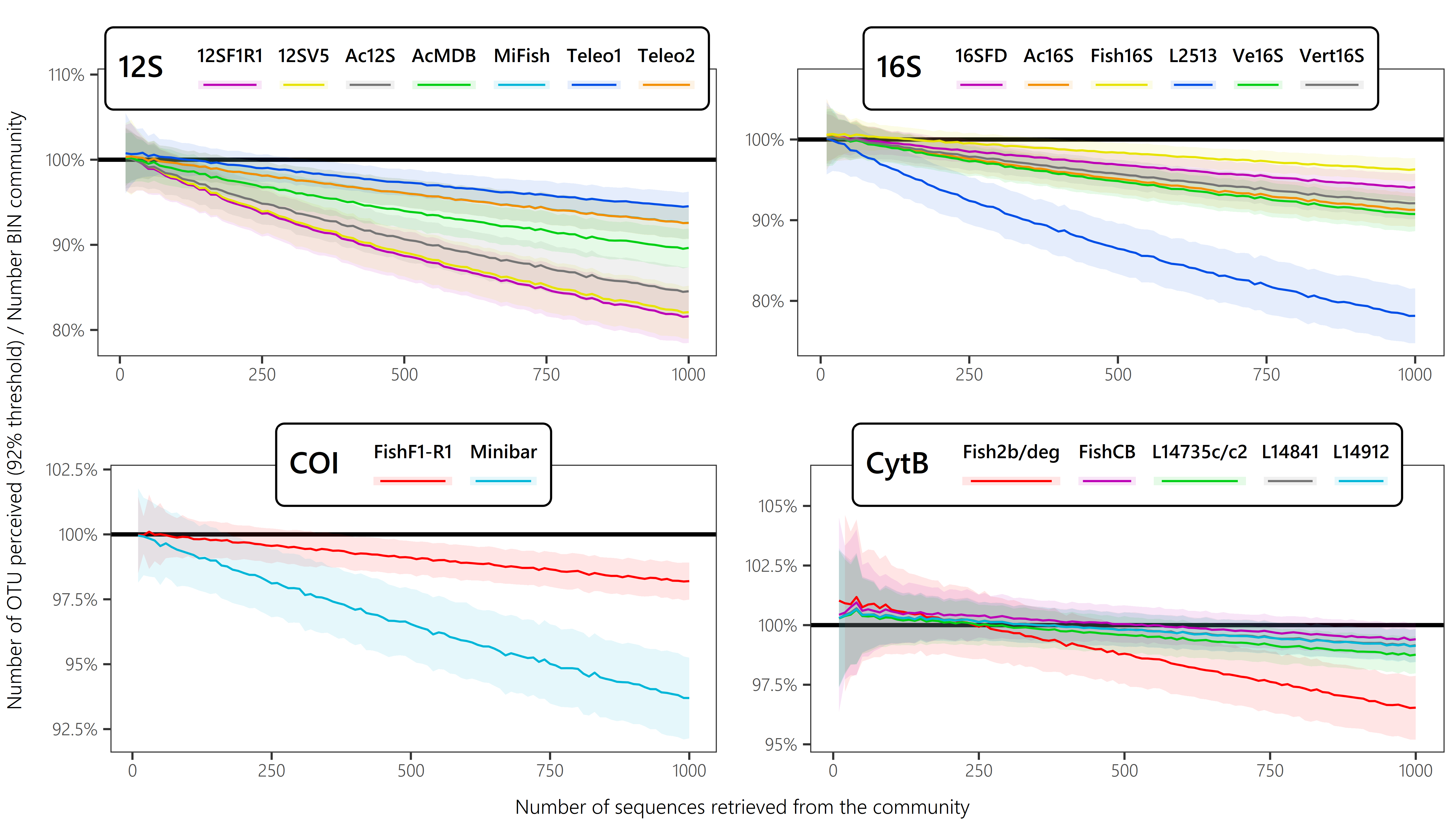


FIGURE S19 | Taxonomic resolution assessment using *in silico* mock communities and a 92% similarity threshold. Each line represents the mean ratio between the number of OTU (*Z_OTU_*) perceived and the actual number of BIN (*Y_BIN_*) across the 1,000 iterations per number of eDNA sequences (*Y_SEQ_*) interval, with its standard deviation in shaded color around (see Figure S11 for details about this analysis). Values higher than 100% correspond to an overestimation of diversity and conversely for values lower than 100%.


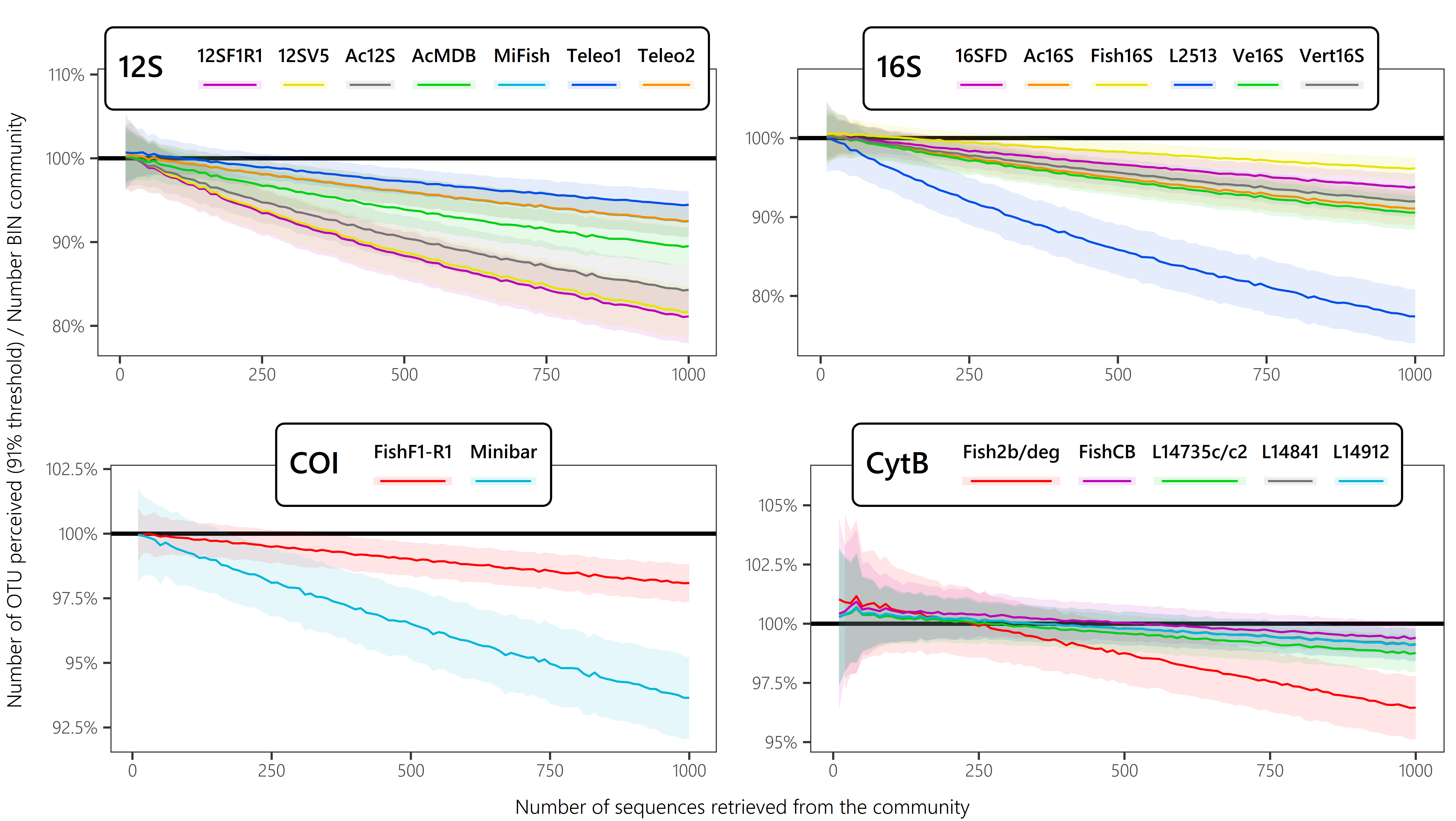


FIGURE S20 | Taxonomic resolution assessment using *in silico* mock communities and a 91% similarity threshold. Each line represents the mean ratio between the number of OTU (*Z_OTU_*) perceived and the actual number of BIN (*Y_BIN_*) across the 1,000 iterations per number of eDNA sequences (*Y_SEQ_*) interval, with its standard deviation in shaded color around (see Figure S11 for details about this analysis). Values higher than 100% correspond to an overestimation of diversity and conversely for values lower than 100%.


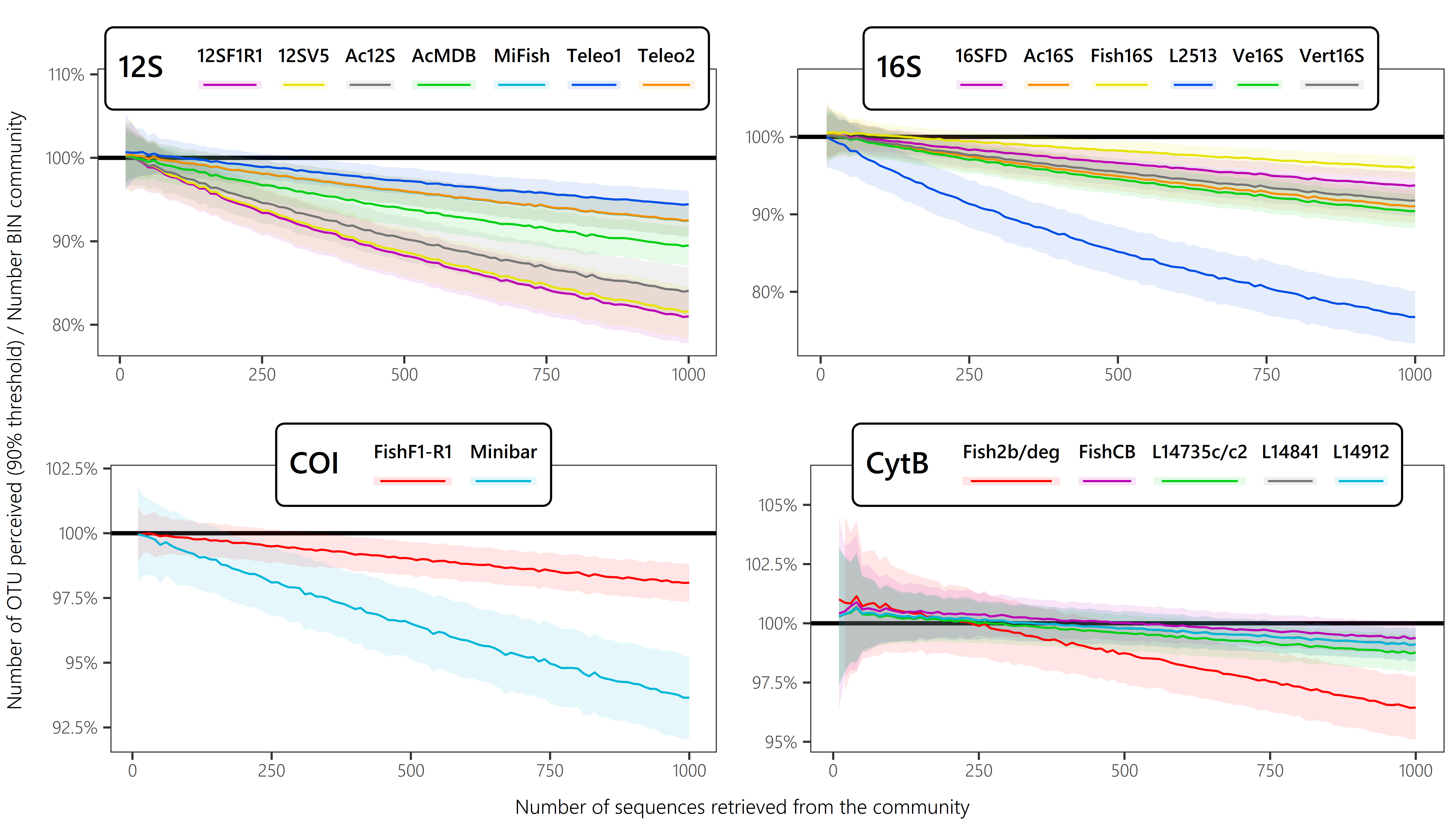


FIGURE S21 | Taxonomic resolution assessment using *in silico* mock communities and a 90% similarity threshold. Each line represents the mean ratio between the number of OTU (*Z_OTU_*) perceived and the actual number of BIN (*Y_BIN_*) across the 1,000 iterations per number of eDNA sequences (*Y_SEQ_*) interval, with its standard deviation in shaded color around (see Figure S11 for details about this analysis). Values higher than 100% correspond to an overestimation of diversity and conversely for values lower than 100%.
